# Supplementary material for: Roles of Remote and Contact Forces in Epithelial Cell Structure Formation
Source: Biophys J. 2020 Feb 5;118(6):1466–78. doi: 10.1016/j.bpj.2020.01.037 (PMC7091513; doi:10.1016/j.bpj.2020.01.037)
Supplement: Document S1. Supporting Materials and Methods and Figs. S1–S5 [file mmc1.pdf]

Biophysical Journal, Volume *118*

Supplemental Information

Roles of Remote and Contact Forces in Epithelial Cell Structure Formation

Tadashi Nakano, Yutaka Okaie, Yasuha Kinugasa, Takako Koujin, Tatsuya Suda, Yasushi Hiraoka, and Tokuko Haraguchi

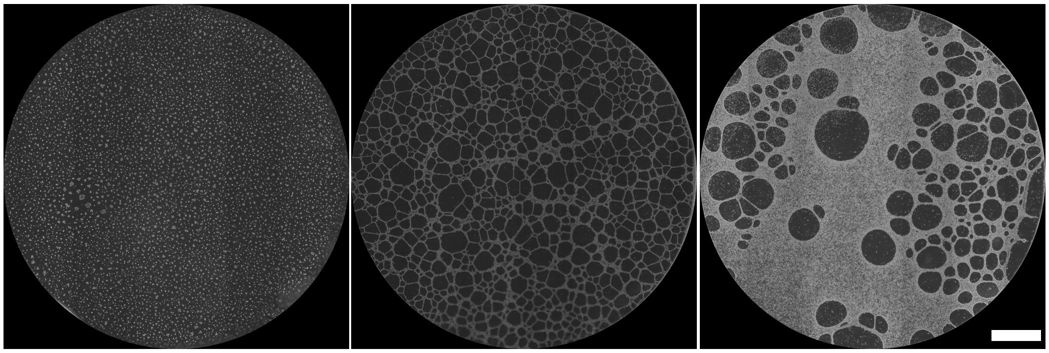

Figure S1. Cell-density dependent structure formation of HT1080 cells (malignant mesenchymal tumor cells) in experiments. Cells were plated on a thick Matrigel layer, and phase-contrast images of the Matrigel area of 14 mm of diameter were taken at 24 h after cells were plated. Similarly to HeLa cells (Fig. 4), HT1080 cells formed islands when the cell density is 200 cells/mm^2 (N = 3), a network-like structure when the cell density is 600 cells/mm^2 (N = 3), and a continent when the cell density is 1100 cells/mm^2 (N = 1). Scale bar, 2 mm.

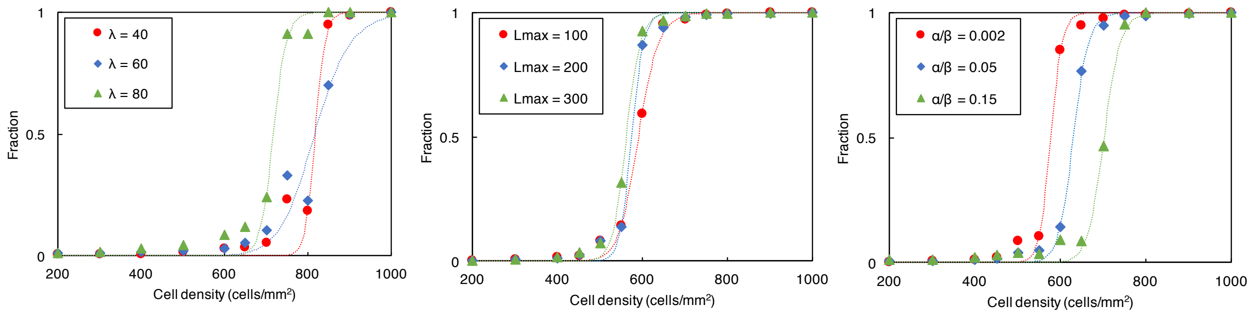

Figure S2. Simulation results on the fraction of cells in the largest cluster as a function of cell density, *(left)* when only the remote force is enabled (α = 0.25 μm/min), *(center)* when only the contact force is enabled (β = 5 μm/min), and *(right)* when both forces are enabled (α is varied, β = 5 μm/min, λ = 40 μm, $L_{\max}$ = 200 μm), respectively. The simulated area is $2R$ = 8 mm. The structure of cells formed at 24 h is examined. Plots represent simulation results, and dotted curves are obtained by fitting the simulation results to the Hill function. The critical cell densities are *(left)* 820, 820, and 720 cells/mm^2 when λ = 40, 60, and 80 μm, *(center)* 590, 570, and 560 cells/mm^2 when $L_{\max}$ = 100, 200, and 300 μm, *(right)* 580, 630, and 700 cells/mm^2 when α/β = 0.002, 0.05, and 0.15, respectively.

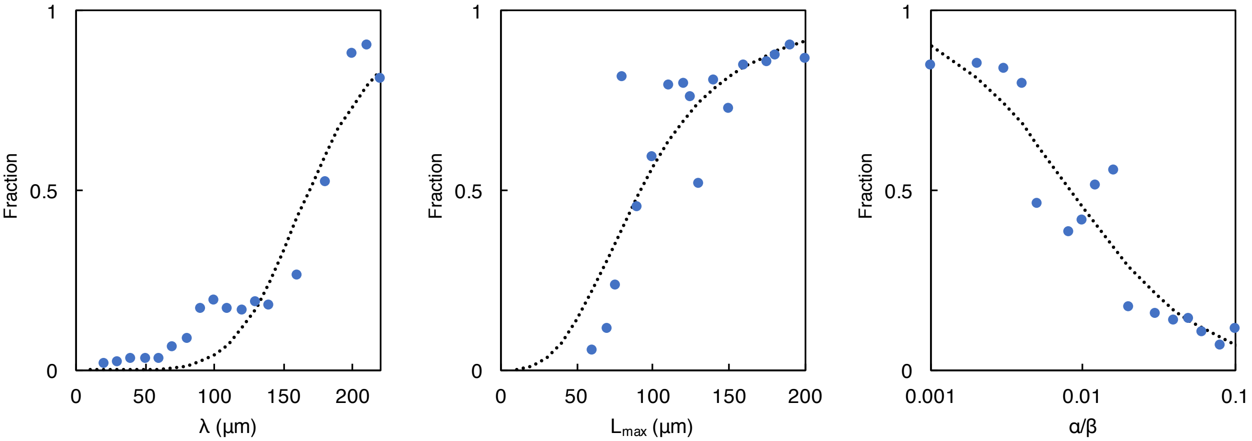

Figure S3. Simulation results on the fraction of cells in the largest cluster *(left)* as a function of λ when only the remote force is enabled (α = 0.25 μm/min), *(center)* as a function of $L_{\max}$ when only the contact force is enabled (β = 5 μm/min), and *(right)* as a function of $\frac{\alpha}{\beta}$ when both forces are enabled (β = 5 μm/min, λ = 40 μm, $L_{\max}$ = 200 μm). The simulated area is $2R$ = 8 mm. ρ = 600 cells/mm^2. The structure of cells at 24 h is examined. Plots represent simulation results, and dotted curves are obtained by fitting the simulation results to the Hill function in the form of $\frac{V^q}{V^q+K^q}$ (left, center) or $\frac{K^q}{V^q+K^q}$ (right), where V is a model parameter (λ, $L_{\max}$ or α/β), q is a coefficient and and K is the critical parameter value. The values of K are *(left)* 170 μm for λ, *(center)* 90 μm for $L_{\max}$, and *(right)* 0.008 for α/β, respectively.

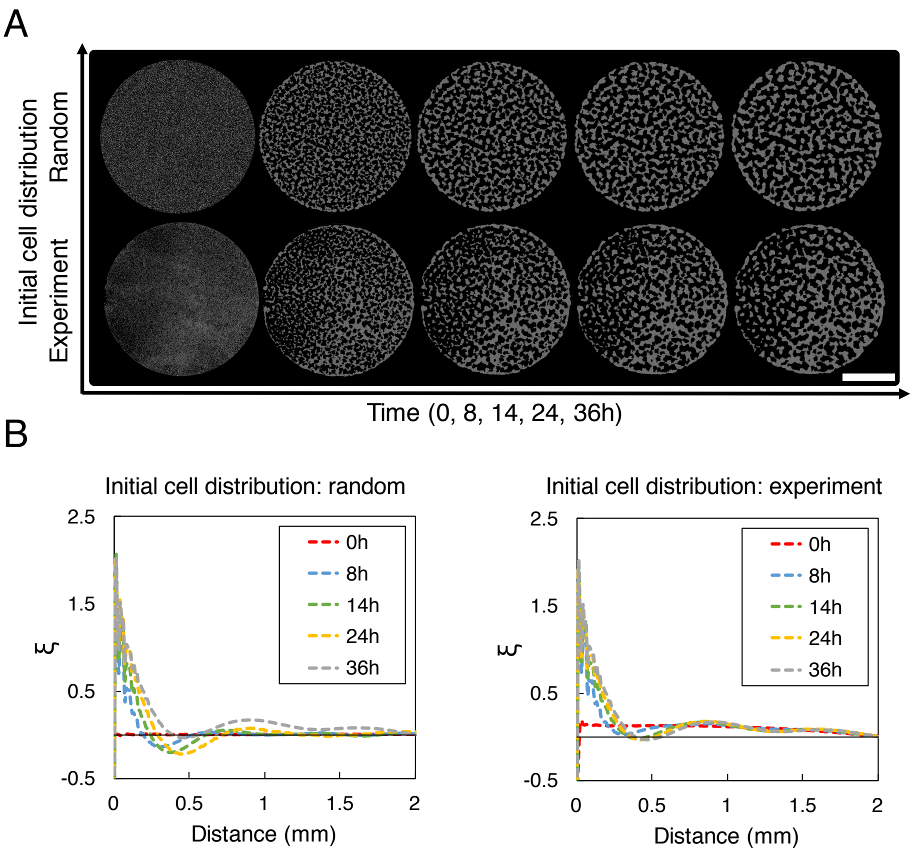

Figure S4. The structures of cells in simulations. *(A)* The structures that cells formed in simulations at 0, 8, 14, 24, and 36 h. α = 0.25 μm/min, λ = 80 μm, β = 5 μm/min, and L_{max} = 200 μm. The initial cell distribution is obtained from a Poisson point process (random) or the experiment (Fig. 4A, network-like). The cell density is approximately ρ = 600 cells/mm^2. Simulated area of $2R$ = 14 mm in diameter is shown. Scale bar, 5 mm. *(B)* Two-point correlation function ξ of cell positions at different time instance for each of the two cases in *(A)*.

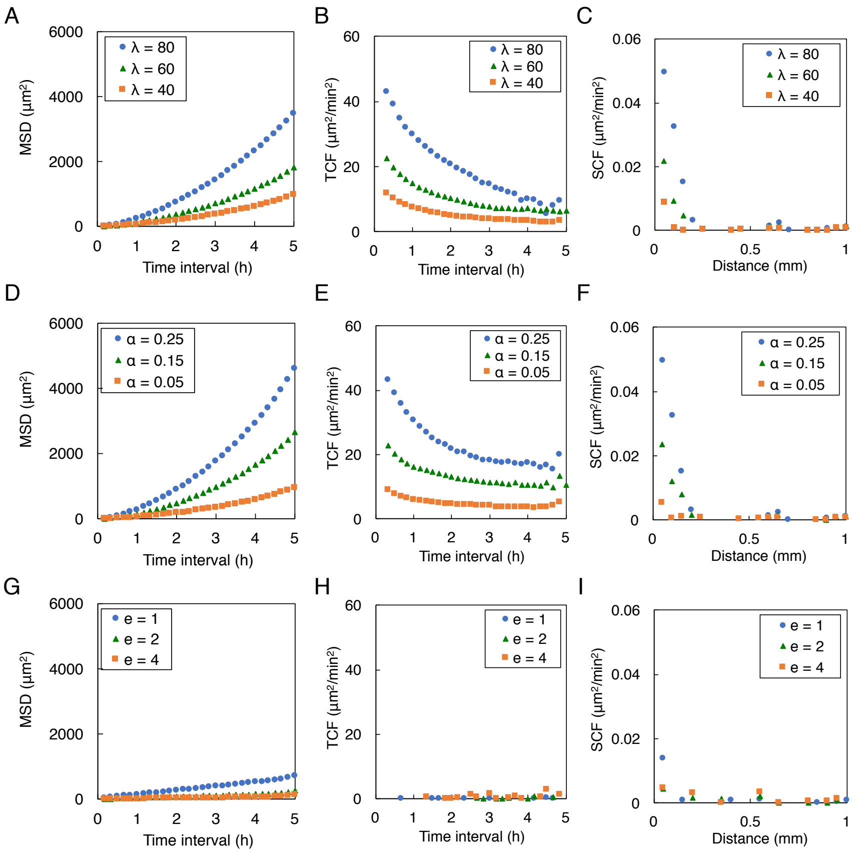

Figure S5. Simulation results. (A, D, G) Mean square displacement (MSD), (B, E, H) temporal correlation function (TCF) of cell velocities, and (C, F, I) spatial correlation function (SCF) of cell velocities. The simulated area is $2R = 4$ mm. The following parameter values were used. (A – C) $\alpha = 0.25$ μm/min, $\lambda = 40, 60, 80$ μm, $\beta = 5$ μm/min, and $L_{\mathrm{max}} = 200$ μm. (D – F) $\alpha = 0.05, 0.15, 0.25$ μm/min, $\lambda = 40$ μm, $\beta = 5$ μm/min, and $L_{\mathrm{max}} = 200$ μm. (G – I) $\alpha = \beta = 0$ μm/min, and $e = 1, 2, 4$. Default values were used for other parameters. The simulations ran over a time period of 5 h. In computing MSD, TCF and SCF, cells within $0.9 \times 2R$ in diameter from the center of the simulation area were used to reduce the effect of boundaries. See text below for details.

Experimental results (Fig. 1 C – E, gel (thick) and gel (thin)) showed that the mean square displacement (MSD), temporal correlation function (TCF) of cell velocities and spatial correlation function (SCF) of cell velocities significantly decreased when the Matrigel thickness reduced from thick (0.65 mm) to thin (0.13 mm). As we discussed in Discussion, we hypothesize that HeLa cells interacted with Matrigel, generated a remote force that propagated over the Matrigel, and coordinated their movements with other cells at distance. We also hypothesize that the Matrigel thickness determined the strength of the remote force and the distance over which the remote force travels over the Matrigel; when the Matrigel was thinner, the remote force was weaker and traveled over a shorter distance. To examine our hypotheses, we decreased the strength (α) and the characteristic length (λ) of the remote force in simulations and examined the impact of these two parameters on MSD, TCF and SCF. Simulation results (Fig. S5 A – F) show that MSD, TCF and SCF decrease as α and λ decrease (namely, the Matrigel thickness decreases), reproducing the behaviors of the decreased MSD, TCF and SCF observed in experiments when the Matrigel thickness decreased (Fig. 1 C – E, gel (thick) and gel (thin)).

Experimental results (Fig. 1 C – E, glass) also showed that, when cells were on a glass surface, MSD increased slowly, while TCF and SCF remained low over time and distance, respectively. As reported previously (28), the Langevin equation describes cell motility on the glass surface. By ignoring the inertial effects in the Langevin equation, and assuming that the remote and contact forces are negligible and, thus, the random force $\mathbf{F}^i_{\mathrm{rnd}}$ being the sole force that acts on cells, we replace our model (5) with the following model: $d\mathbf{x}_i/dt = \mathbf{F}^i_{\mathrm{rnd}}$, where $\mathbf{F}^i_{\mathrm{rnd}}$ follows a Gaussian distribution with zero mean and the variance of e^2 for each dimension. Note that the volume exclusion effect remained effective in simulations. Simulation results (Fig. S5 G – I) with the values of e varied ($e = 1, 2, 4$) show MSD, TCF and SCF exhibiting the behaviors similar to those observed in experiments (Fig. 1 C – E, glass), respectively.

Movies

Movie S1 5-h time-lapse movie when cells were plated on a glass surface (Fig. 1A and B, glass)

Movie S2 5-h time-lapse movie when cells were plated on a thin Matrigel layer [Fig. 1A and B, gel (thin)]

Movie S3 5-h time-lapse movie when cells were plated on a thick Matrigel layer [Fig. 1A and B, gel (thick)]

Movie S4 15-h time-lapse movie when cells were plated on a thick Matrigel layer (Fig. 2A and B)

Movie S5 5-h time-lapse movie showing cells forming cellular bridges (Fig. 3A)

Movie S6 Simulation of the cellular bridge formation observed in experiments (Fig. 7A)

Movie S7 Simulation of large-scale multicelluarlr structure formation (Fig. 8A, islands)

Movie S8 Simulation of large-scale multicellualr structure formation (Fig. 8A, network-like)

Movie S9 Simulation of large-scale multicellualr structure formation (Fig. 8A, continent)

Numerical Methods

Discrete-time and agent-based simulations were performed in this work. Time t is discretized with the time step length of Δt, and each simulation step advances time by Δt. Each cell $i \in \mathcal{N}$ is modeled as a discrete entity or an agent.

Simulations consider a set $\mathcal{N}$ of cells in a two-dimensional and circular area of the diameter of $2R$. At time t, cell i maintains its position $\mathbf{x}_i(t)$ and list $\mathcal{N}_i(t)$ containing cells that are in physical contact with cell i. Cell i also maintains its Verlet Neighbor List (VNL) $\mathcal{V}_i(t)$ containing cells that are within the distance L_{vnl} from cell i, excluding cell i itself. VNL is used to facilitate the computation of the remote force between cells. A simulation runs in the following manner:

1. Initialization Initial positions of cells, $\mathbf{x}_i(0)$ for all $i \in \mathcal{N}$, are set following a Poisson point process or based on the cell positions observed in experiments. For each cell $i \in \mathcal{N}$, its initial physical contact list $\mathcal{N}_i(0)$ and initial VNL $\mathcal{V}_i(0)$ are then set such that

- $\mathcal{N}_i(0)$ contains all cells within the contact initiation distance L_{ini} from cell i, excluding cell i itself, and
- $\mathcal{V}_i(0)$ contains all cells within the distance L_{vnl} from cell i, excluding cell i itself.

2. Main loop At every simulation time step, for each cell $i \in \mathcal{N}$, its position $\mathbf{x}_i(t)$ is updated as follows. When no cells exist within distance $L_{\min}$ from $\mathbf{x}_i(t)$,

$$\mathbf{x}_i(t+\Delta t) = \mathbf{x}_i(t) + \left(\mathbf{F}_i^{\text{rm}}(t) + \mathbf{F}_i^{\text{cn}}(t)\right)\Delta t, \tag{10}$$

and otherwise,

$$\mathbf{x}_i(t+\Delta t) = \mathbf{x}_i(t) + \mathbf{F}_i^{\text{ex}}(t)\Delta t, \tag{11}$$

where $\mathbf{F}_i^{\text{rm}}(t)$ and $\mathbf{F}_i^{\text{cn}}(t)$ represent the remote and contact forces exerted on cell i at time t, respectively, and $\mathbf{F}_i^{\text{ex}}(t)$ represents the effect of volume exclusion that acts on cell i at time t. These three terms $\mathbf{F}_i^{\text{rm}}(t)$, $\mathbf{F}_i^{\text{cn}}(t)$ and $\mathbf{F}_i^{\text{ex}}(t)$ are computed as follows.

$$\mathbf{F}_i^{\text{rm}}(t) = \alpha \sum_{\substack{j \in \mathcal{V}_i(t) \\ \left|\mathbf{x}_i(t)-\mathbf{x}_j(t)\right| \le L_{\text{cut}}}} \exp\left(-\frac{\left|\mathbf{x}_j(t)-\mathbf{x}_i(t)\right|}{\lambda}\right) \frac{\mathbf{x}_j(t)-\mathbf{x}_i(t)}{|\mathbf{x}_j(t)-\mathbf{x}_i(t)|}, \tag{12}$$

$$\mathbf{F}_i^{\text{cn}}(t) = \beta \sum_{j \in \mathcal{N}_i(t)} \max\left(\frac{\left|\mathbf{x}_j(t)-\mathbf{x}_i(t)\right| - L_{\min}}{L_{\max} - L_{\min}}, 0\right) \frac{\mathbf{x}_j(t)-\mathbf{x}_i(t)}{|\mathbf{x}_j(t)-\mathbf{x}_i(t)|}, \tag{13}$$

$$\mathbf{F}_i^{\text{ex}}(t) = -\gamma \sum_{j \in \mathcal{N}_i(t)} \max\left(\frac{L_{\min} - \left|\mathbf{x}_j(t)-\mathbf{x}_i(t)\right|}{L_{\min}}, 0\right) \frac{\mathbf{x}_j(t)-\mathbf{x}_i(t)}{|\mathbf{x}_j(t)-\mathbf{x}_i(t)|} \tag{14}$$

Note that in calculating the remote force in Eq. 12, only the cells that are within the remote force cutoff distance L_{cut} of each other are considered. This is to avoid incurring additional computation time. In our simulations, we set $L_{\text{cut}} = 10\lambda$ and $L_{\text{vnl}} = 12\lambda$. However, these values may need to be decreased for computational tractability, especially when the cell density is very high.

In addition, for each cell $i \in \mathcal{N}$, its contact list $\mathcal{N}_i(t)$ is updated at every simulation time step as follows.

$$\mathcal{N}_i(t+\Delta t) = \mathcal{N}_i(t) \cup \mathcal{E}_i^+(t) \setminus \mathcal{E}_i^-(t), \tag{15}$$

where $\mathcal{E}_i^+(t)$ is the set of new cells with which cell i becomes in physical contact at time t, and $\mathcal{E}_i^-(t)$ is the set of cells with which cell i loses physical contact at time t. They are given below:

$$\mathcal{E}_i^+(t) = \left\{j : j \in \mathcal{V}_i(t) \setminus \mathcal{N}_i(t), \left|\mathbf{x}_i - \mathbf{x}_j\right| \le L_{\text{ini}}, i \ne j\right\}, \tag{16}$$

$$\mathcal{E}_i^-(t) = \left\{j : j \in \mathcal{N}_i(t), \left|\mathbf{x}_i - \mathbf{x}_j\right| > L_{\max}, i \ne j\right\}. \tag{17}$$

Further, for each cell $i \in \mathcal{N}$, we update at every 100 simulation time steps its $\mathcal{V}_i(t)$ such that $\mathcal{V}_i$ contains all cells within L_{vnl} from cell i, excluding cell i itself. $\mathcal{V}_i(t)$ is given as follows.

$$\mathcal{V}_i(t) = \left\{j : j \in \mathcal{N}, \left|\mathbf{x}_i(t) - \mathbf{x}_j(t)\right| < L_{\text{vnl}}, i \ne j\right\} \tag{18}$$

Note that $\mathcal{V}_i(t)$ is not updated at every simulation time step to avoid incurring additional computation time.

Obtaining Two Point Correlation Functions

We computed the two-point correlation function $\xi(r)$ in Eq. 4, also shown below,

$$\mathrm{d}N(r) = 2\pi r \mathrm{d}r \rho \left(1 + \xi\left(r\right)\right), \tag{19}$$

in the following manner.

The left-hand side of Eq. 19, $\mathrm{d}N(r)$, represents the number of cells located at distance r to $\mathrm{d}r$ from a randomly selected cell in the circular area of experiments or simulations. Computing $\mathrm{d}N(r)$ requires the spatial positions of all cells. To compute $\mathrm{d}N(r)$ from experimental results, we used the spatial positions of cells identified from microscopy images of cells. To compute $\mathrm{d}N(r)$ from simulations, we used the spatial positions of all cells tracked in simulations. Once $\mathrm{d}N(r)$ is computed, $\xi(r)$ can be obtained using Eq. 19.

In computing $\mathrm{d}N(r)$, and thus, $\xi(r)$, in Eq. 19, we reduced the effect of boundaries of the Matrigel area in experiments and of the simulated area as follows. We first selected a subset $\mathcal{N}'$ of cells such that each cell $i \in \mathcal{N}'$ is located within 0.4 mm from the center of the area when $R = 4$ mm or 2.8 mm from the center of the area when $R = 7$ mm. We then counted $\mathrm{d}N(r)$, varying r from 0 to 2.8 mm with an increment of $\mathrm{d}r = 0.02$ mm as follows:

$$\mathrm{d}N(r) = \frac{1}{|\mathcal{N}'|} \sum_{i \in \mathcal{N}'} \left| \left\{ j : j \in \mathcal{N}, r < d_{i,j} \leq r + \mathrm{d}r \right\} \right|, \tag{20}$$

where $d_{i,j}$ is the distance between cells i and j.
