# Supplementary material for: Roles of Remote and Contact Forces in Epithelial Cell Structure Formation
Source: Biophys J. 2020 Feb 5;118(6):1466–78. doi: 10.1016/j.bpj.2020.01.037 (PMC7091513; doi:10.1016/j.bpj.2020.01.037)
Supplement: Document S2. Article plus Supporting Material [file mmc11.pdf]

Biophysical Journal
Article

Biophysical Society

Roles of Remote and Contact Forces in Epithelial Cell Structure Formation

Tadashi Nakano,[1,*] Yutaka Okaie,[1] Yasuha Kinugasa,[2] Takako Koujin,[3] Tatsuya Suda,[4] Yasushi Hiraoka,[2,3] and Tokuko Haraguchi[2,3]

[1]Institute for Datability Science, Osaka University, Suita, Japan; [2]Graduate School of Frontier Biosciences, Osaka University, Suita, Japan; [3]Advanced ICT Research Institute Kobe, National Institute of Information and Communications Technology, Kobe, Japan; and [4]University Netgroup, Fallbrook, California

ABSTRACT Cancer cells collectively form a large-scale structure for their growth. In this article, we report that HeLa cells, epithelial-like human cervical cancer cells, aggressively migrate on Matrigel and form a large-scale structure in a cell-density-dependent manner. To explain the experimental results, we develop a simple model in which cells interact and migrate using the two fundamentally different types of force, remote and contact forces, and show how cells form a large-scale structure. We demonstrate that the simple model reproduces experimental observations, suggesting that the remote and contact forces considered in this work play a major role in large-scale structure formation of HeLa cells. This article provides important evidence that cancer cells form a large-scale structure and develops an understanding into the poorly understood mechanisms of their structure formation.

SIGNIFICANCE Recent experimental studies show increasing evidence that cancer cells form a large-scale structure, specifically a vascular-like structure, allowing cancer cells to gain access to blood vessels and nutrient sources in a cooperative manner (1–5). Little is known, however, about how cancer cells form such a structure. In this article, we provide important empirical evidence of large-scale structure formation of cancer cells. We also develop a simple deterministic model to understand how cancer cells form a large-scale structure. The insights obtained from this work will contribute future development of cancer research and medicine.

INTRODUCTION

How cells interact and form a large-scale structure is a long-standing question in biology. Bacteria form biofilms and obtain increased resistance to antimicrobial agents (6,7); epithelial cells form monolayers and protect the tissues that lie beneath from radiation, desiccation, toxins, invasion by pathogens, and physical trauma (8); endothelial cells form capillaries and convey blood between veins and arteries (9,10); cancer cells form a vascular-like structure to gain access to blood vessels and nutrient sources (4,5); and acellular slime molds form a tubular network and distribute nutrients within themselves (11). A key to understanding how cells interact and form a large-scale structure is to identify major forces that act between cells and develop a simple model based on such forces that captures underlying biochemical and biophysical details.

When cells form a large-scale structure, two fundamentally different types of force play a role: remote and contact forces. The remote force refers to the force that one cell exerts on another at a distance, whereas the contact force is the one that acts between two cells in physical contact. An example of the remote force is the chemotactic force. Human umbilical vein endothelial cells have been proposed to secrete vascular endothelial growth factors to create the vascular endothelial growth factor gradient in their environment and attract other cells at a distance (9). Another example of the remote force is the haptotactic force. Human umbilical vein endothelial cells and human microvascular endothelial cells directionally move on a surface of an adhesive substrate, such as the extracellular matrix (ECM), according to the adhesion gradient or the gradient of surface-bound molecules (12,13). Yet another example of the remote force is the mechanotactic force. Vascular endothelial cells mechanically deform the ECM to change

Submitted September 13, 2019, and accepted for publication January 29, 2020.

*Correspondence: tnakano@ids.osaka-u.ac.jp

Editor: Ruth Baker.

https://doi.org/10.1016/j.bpj.2020.01.037

© 2020 Biophysical Society.

This is an open access article under the CC BY license (http://creativecommons.org/licenses/by/4.0/).

the ECM rigidity and attract other cells at a distance (14–18). Note that the term "remote force" reflects that a cell exerts the force on another cell remotely from a distance without making physical contact and that our model avoids explicitly modeling an underlying physical mechanism that induces the force—for instance, diffusive chemoattractants in the chemotactic force. On the other hand, the contact force is commonly observed with many cell types. It includes the force that is mediated through cadherin-dependent cell-cell adhesion: cells use this attraction force, adhere to each other, and collectively migrate (19,20). The contact force also includes the attraction force that is mediated through cellular bridges formed between cells: human bronchial epithelial cells physically connect with each other by forming bridges and migrate toward each other (21,22).

The purpose of this study is to understand large-scale structure formation of HeLa cells (human cervical cancer cells). We first report that HeLa cells, which are relatively nonmotile on glass surfaces, aggressively move on Matrigel, a gelatinous protein mixture resembling the extracellular environment in tissues (23), and form a large-scale structure in a cell-density-dependent manner. We then present a simple model of cell migration considering remote and contact forces and show that our model can reproduce experimental observations.

MATERIALS AND METHODS

Cell culture

HeLa cells, originally derived from cervical cells taken from Henrietta Lacks, were obtained from the Riken Cell Bank (Tsukuba, Japan). HT1080 cells were obtained from the American Type Culture Collection (Manassas, Virginia). Cells were maintained in standard culture dishes in Dulbecco's Modified Eagle Medium supplemented with 10% fetal calf serum at 37°C under 5% CO_2.

Preparation of samples

A layer of Matrigel (8–12 mg/mL; Falcon) was formed in 35-mm glass-bottom dishes (Matsunami Glass, Kishiwada, Japan) for structure formation experiments. 20 or 100 μL of Matrigel was added to a circular well of 14 mm in diameter in each dish to form either a thin layer or a thick layer of Matrigel. Based on the information provided in the Matrigel manufacture's manual, thin and thick Matrigel layers are estimated to be 0.13 and 0.65 mm in thickness. The dishes were then incubated for 30 min at 37°C to allow the Matrigel to gel, and cells were plated on the Matrigel and maintained under the same cell culture conditions as those described above. The cell density was varied from 100 to 1100 cells/mm^2. In control experiments, Matrigel was not used.

Time-lapse imaging experiments

For time-lapse imaging, HEPES (final concentration, 20 mM, pH 7.3) was added to the cell culture medium of the prepared samples to avoid an increase in pH during experiments. A layer of mineral oil was also overlaid on top of the medium to avoid evaporation of the medium during experiments. The Olympus IX83 inverted microscope with a 4$\times$ or 10$\times$ objective lens and the Olympus DP80 CCD camera were used to collect phase-contrast images of cells every 30 s for up to 24 h. Experiments were performed at 37°C in a temperature-controlled room.

Cell migration analysis

Cell trajectories were obtained from time-lapse images for cell migration analysis. Time in experiments was divided into four nonoverlapping time segments of 0–5, 5–10, 10–15, and 15–20 h to examine the time-variant migration behavior of cells. For each time segment, MTrackJ (24) was used to obtain trajectories of M cells; cell i's trajectory $(i = 1, 2, \cdots, M)$ contains a series of its positions in two-dimensional space observed at the time interval of $\Delta t = 10$ min in each time segment of 5 h.

For each time segment, we computed the mean-square displacement (MSD), defined as

$$\mathrm{MSD}(\tau) = \left\langle |\mathbf{x}_i(t+\tau) - \mathbf{x}_i(t)|^2 \right\rangle, \tag{1}$$

where $\mathbf{x}_i(t)$ is cell i's position at time t, τ is the time lag ($\tau = \Delta t, 2\Delta t, \cdots$), and the average $\langle \cdots \rangle$ is taken over all cells and over all instances of time in the 5-h time segment.

We also computed the temporal correlation function (TCF) and spatial correlation function (SCF) of cell velocities. TCF is defined as

$$\mathrm{TCF}(\tau) = \langle \mathbf{v}_i(t) \cdot \mathbf{v}_i(t+\tau) \rangle, \tag{2}$$

where $\mathbf{v}_i(t) = (\mathbf{x}_i(t+\Delta t) - \mathbf{x}_i(t))/\Delta t$ is cell i's velocity at time t, $\tau\ (= 2\Delta t, 3\Delta t, \cdots)$ is the time lag, and the average $\langle \cdots \rangle$ is taken over all cells and all time instances in the 5-h time segment. SCF is defined as

$$\mathrm{SCF}(r) = \left\langle \mathbf{v}_i(t) \cdot \mathbf{v}_j(t) \right\rangle, \tag{3}$$

where the average $\langle \cdots \rangle$ is taken over all pairs of cells (i and j) that are separated by distance r and over all instances of time in the 5-h time segment.

Large-scale multicellular structure analysis

The large-scale structure of cells was quantified using the two-point correlation function $\xi(r)$, which is defined below:

$$\mathrm{d}N(r) = 2\pi r \mathrm{d}r \rho (1 + \xi(r)), \tag{4}$$

where $\mathrm{d}N(r)$ is the number of cells located between distance r and $r + \mathrm{d}r$ from a randomly chosen cell, and ρ is the average cell density. When a set $\mathcal{N}$ of cells exists in a circular area of diameter $2R$, $\rho = (|\mathcal{N}|/\pi R^2)$. The two-point correlation function $\xi(r)$ indicates the degree to which the number of cells between distance r and $r + \mathrm{d}r$ from a randomly chosen cell differs from that of randomly distributed cells. When cells are randomly distributed following a Poisson point process, $\xi(r) = 0$; when more cells are found between distance r and $r + \mathrm{d}r$ than randomly distributed cells, $\xi(r) > 0$; and when less cells are found between distance r and $r + \mathrm{d}r$, $\xi(r) < 0$. The two-point correlation function is often used to characterize the spatial distribution of galaxies in the universe (25).

Cell migration model

To understand how HeLa cells form a large-scale structure using the remote and contact forces, we develop a simple cell migration model that incorporates these forces. We consider a set $\mathcal{N}$ of cells in a two-dimensional circular space of radius R. For simplicity, the total number $|\mathcal{N}|$ of cells is constant over time in our model.

For cell $i \in \mathcal{N}$, we apply Newton's second law, neglecting its inertial effects (26,27), and describe the governing equation as follows:

$$\frac{d\mathbf{x}_i}{dt} = \mathbf{F}_i^{\mathrm{rm}} + \mathbf{F}_i^{\mathrm{cn}}, \tag{5}$$

where $\mathbf{x}_i$ is cell i's position at time t. $\mathbf{F}_i^{\mathrm{rm}}$ and $\mathbf{F}_i^{\mathrm{cn}}$ represent the remote and contact forces, respectively. Individual models of cell migration (28–33) often use the Langevin equation and are stochastic with random motion, whereas our model is deterministic and simpler than these models.

Here, we first obtain the expression for $\mathbf{F}_i^{\mathrm{rm}}$. As explained earlier, examples of the remote force include chemotactic and mechanotactic forces. These forces are mediated through a force field that cells create. In chemotaxis, a cell emits chemoattractants, creates a concentration gradient (i.e., a force field) of the chemoattractants in its environment, and attracts other cells at a distance. In mechanotaxis, a cell mechanically changes the ECM rigidity, creates a gradient (i.e., a force field) of the ECM rigidity in its environment, and attracts other cells at a distance.

Let $c(r)$ denote the force field at distance r from the cell that created the force field. In obtaining the expression for $\mathbf{F}_i^{\mathrm{rm}}$, we assume that the rate of change in $c(r)$ is given by a linear diffusion equation in an isotropic and homogeneous environment and that $c(r)$ decays at a constant rate. We further assume that $c(r)$ reaches its equilibrium instantaneously because cell movement in our experiments was significantly slower than the propagation of the remote force or force-carrying particles, such as chemoattractants (34). Using these assumptions, we obtain the following expression of $c(r)$:

$$c(r) \propto \exp\left(-\frac{r}{\lambda}\right), \tag{6}$$

where λ (>0) is the characteristic length of the exponential decay (35–37). Under these assumptions, $\mathbf{F}_i^{\mathrm{rm}}$ is given as follows:

$$\mathbf{F}_i^{\mathrm{rm}} = \alpha \sum_{j \in \mathcal{N}, j \neq i} \exp\left(-\frac{|\mathbf{x}_j - \mathbf{x}_i|}{\lambda}\right) \frac{\mathbf{x}_j - \mathbf{x}_i}{|\mathbf{x}_j - \mathbf{x}_i|}, \tag{7}$$

where α is a positive constant and determines the strength of the remote force that cell i receives from all other cells. The summation in Eq. 7 shows that each and every cell, excluding cell i itself, pulls cell i to its direction and contributes to $\mathbf{F}_i^{\mathrm{rm}}$, which acts on cell i. Note that Eq. 7 represents an exponentially decaying force that propagates in all directions; this expression is simple but general and not limited to the examples of the remote force given earlier.

We next obtain the expression for $\mathbf{F}_i^{\mathrm{cn}}$. In obtaining the expression for $\mathbf{F}_i^{\mathrm{cn}}$, we assume the following. Two cells i and j make physical contact and form cell-cell adhesion when they first come within the contact initiation distance L_{ini} to each other. Once cells make physical contact, they maintain the contact while they move within the contact termination distance $L_{\max}$ to each other. When cell j is within the distance range of $[L_{\min}, L_{\max}]$ to cell i, where $L_{\min}(<L_{\mathrm{ini}})$ is the minimal cell-to-cell distance allowed, cell j pulls cell i toward itself with the strength that is proportional to the distance to cell i. When they move away from each other and become farther apart than $L_{\max}(>L_{\mathrm{ini}})$, they lose their physical contact. By applying simple linear elasticity, $\mathbf{F}_i^{\mathrm{cn}}$ is given as follows:

$$\mathbf{F}_i^{\mathrm{cn}} = \beta \sum_{j \in \mathcal{N}_i} \max\left(\frac{|\mathbf{x}_j - \mathbf{x}_i| - L_{\min}}{L_{\max} - L_{\min}}, 0\right) \frac{\mathbf{x}_j - \mathbf{x}_i}{|\mathbf{x}_j - \mathbf{x}_i|}, \tag{8}$$

where β is a positive constant and determines the strength of the contact force that cell i receives from all other cells with which cell i is in physical contact. In Eq. 8, $\mathcal{N}_i$ is a set of cells that maintain physical contact with cell i at time t, and $\max(\bullet)$ returns a nonzero (positive) value when the distance from cell i to cell $j \in \mathcal{N}_i$ is longer than $L_{\min}$.

We note that the contact force acts between two cells that are in physical contact, including two cells that made physical contact in the past and continue maintaining it; the contact force has a memory in this regard, unlike cell-cell interaction forces in typical cell migration models (30–32), in which the force is memory-less and acts between two cells based only on their current distance.

The model based on Eq. 5 does not consider the cell size, and it allows multiple cells to occupy the same position. This, however, was rarely observed in our experiments. We therefore implemented a volume exclusion effect (38,39) in the following manner. If there exists cell j ($j \in \mathcal{N}, j \neq i$) within $L_{\min}$ from cell i, the right-hand side of Eq. 5 is replaced with the volume exclusion effect $\mathbf{F}_i^{\mathrm{ex}}$, given below:

$$\mathbf{F}_i^{\mathrm{ex}} = -\gamma \sum_{j \in \mathcal{N}, j \neq i} \max\left(\frac{L_{\min} - |\mathbf{x}_j - \mathbf{x}_i|}{L_{\min}}, 0\right) \frac{\mathbf{x}_j - \mathbf{x}_i}{|\mathbf{x}_j - \mathbf{x}_i|}, \tag{9}$$

where γ is a positive constant that determines the strength of the volume exclusion effect on cell i from all other cells that are within $L_{\min}$ to cell i. In Eq. 9, $\max(\bullet)$ returns a nonzero (positive) value when the distance from cell i to cell j ($j \in \mathcal{N}, j \neq i$) is shorter than $L_{\min}$. The volume exclusion effect $\mathbf{F}_i^{\mathrm{ex}}$ acts in the single-cell size scale ($\sim L_{\min}$) and avoids multiple cells from occupying the same position.

Numerical methods and parameter values

The governing equation (Eq. 5) was solved using the method described in the Supporting Material. The volume exclusion effect was also calculated when cells are located within a distance of $L_{\min}$ from each other. Time was discretized with an interval of $\Delta t = 0.1$ min. To simulate a large number of cells with realistic computing time, we applied a cutoff distance (i.e., the maximal distance over which the remote force acts) and maintained Verlet lists to avoid computing distances for all possible cell pairs at every simulation time step. These techniques are commonly used in molecular dynamics simulations (40) and are applicable to our model because the remote force decays exponentially with distance and because its strength diminishes quickly in our model.

We used the following configurations and parameter values in simulations unless otherwise noted. Cells moved in a circular area of $2R = 8$ mm in diameter or $2R = 14$ mm (the same area size with experiments). The cell density ρ was set as $\rho = 600$ cells/mm^2, at which a network-like structure was formed in experiments. Initial cell positions were determined using a Poisson point process. The minimal cell-cell distance and contact initiation distance were estimated based on experimental observations, and they were $L_{\min} = 25$ μm and $L_{\mathrm{ini}} = 50$ μm. The coefficient α and characteristic length λ of the remote force and the coefficient β and contact termination distance $L_{\max}$ of the contact force were unknown and varied in simulations. The coefficient of volume exclusion effect was set to allow two overlapping cells to move to nonoverlapping positions in one simulation time step (i.e., $\gamma = L_{\min}/\Delta t$).

RESULTS

HeLa cells increase motility on Matrigel

HeLa cells showed increased motility in the first 5 h of experiments when cultured on Matrigel. In the control experiments using a glass surface without Matrigel, cells stayed at or near their initial positions (Fig. 1, *A* and *B*, glass) and showed a relatively small (1400 μm^2) MSD of their positions over 5 h (Fig. 1 *C*). When cultured on a thin Matrigel

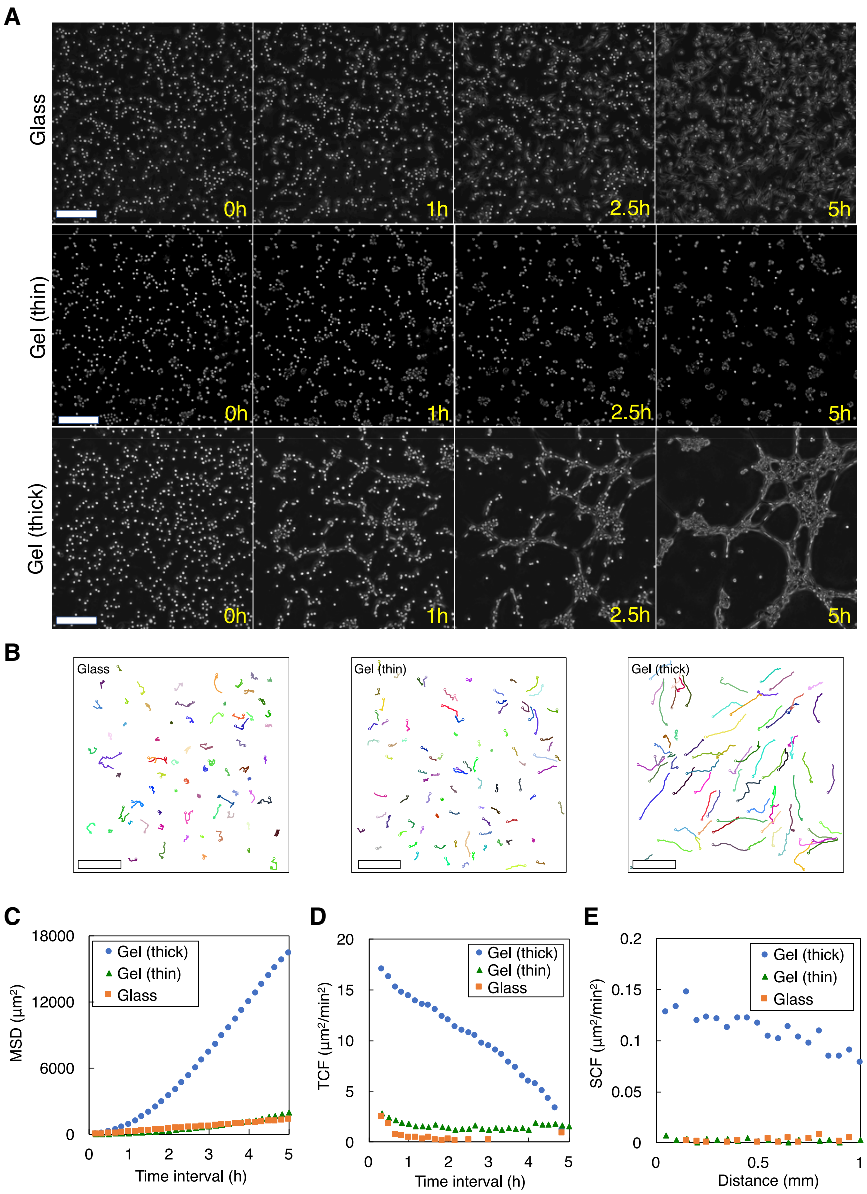

FIGURE 1 HeLa cell motility in the first 5 h of experiments. (*A*) Phase-contrast images of cells on a glass surface, a thin Matrigel layer, and a thick Matrigel layer at 0–5 h after cells were seeded. Approximately 700 cells were identified in each experiment at 0 h. Scale bars, 200 μm. See Videos S1, S2, and S3. (*B*) 5-h cell trajectories on a glass surface ($M = 71$), on a thin Matrigel layer ($M = 88$), and on a thick Matrigel layer ($M = 71$). Scale bars, 200 μm. From the cell trajectories, (*C*) mean-square displacement (MSD), (*D*) temporal correlation function (TCF) of cell velocities, and (*E*) spatial correlation function (SCF) of cell velocities were obtained. To see this figure in color, go online.

layer, HeLa cells slightly increased motility (Fig. 1, *A* and *B*, gel (thin)) and showed a 1.4-fold increase to 2000 μm^2 in their 5-h MSD (Fig. 1 *C*). When cultured on a thick Matrigel layer, HeLa cells significantly increased motility (Fig. 1, *A* and *B*, gel (thick)); the 5-h MSD was 16,400 μm^2 (Fig. 1 *C*), a magnitude larger than those on a glass surface and a thin Matrigel layer.

HeLa cells maintained their moving direction for a longer time period on a Matrigel layer than on a glass surface. On both thin and thick Matrigel layers, the TCF of cell velocities gradually decreased over 5 h (50% decrease in 210 min on a thick Matrigel layer and in 170 min on a thin Matrigel layer), whereas on a glass surface, it decreased quickly (50% decrease in 40 min) (Fig. 1 *D*), showing that cell movements on Matrigel are more persistent and that those on a glass surface are more random. Furthermore, HeLa cells on a thick Matrigel layer clearly exhibited collective motion. As indicated by the SCF of cell velocities (Fig. 1 *E*), cells on a thick Matrigel layer showed highly correlated movement in a relatively large area ($\sim$1 mm), whereas cells on a glass surface and on a thin Matrigel layer showed little such movement.

HeLa cells on a thick Matrigel layer decrease motility after becoming a part of a structure

HeLa cells on a thick Matrigel layer decreased motility in 5–20 h as the cells started aggregating and forming a spatially distinct structure and becoming a part of the structure (Fig. 2, *A* and *B*). The MSD clearly decreased as time

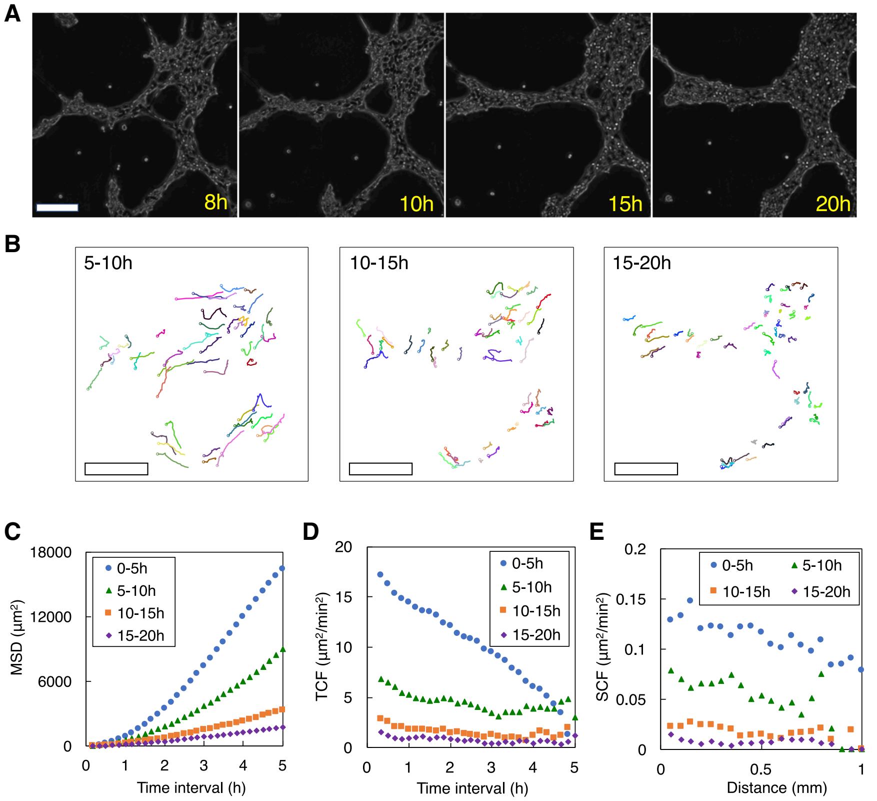

FIGURE 2 HeLa cell motility on a thick Matrigel layer in 5–20 h of experiments. (*A*) Phase-contrast images of cells at 8, 10, 15, and 20 h after cells were seeded. Scale bar, 200 μm. See Video S4. (*B*) Cell trajectories ($M = 53$) in the three time segments of 5–10, 10–15, and 15–20 h. Scale bars, 200 μm. From the cell trajectories, (*C*) mean-square displacement (MSD), (*D*) temporal correlation function (TCF) of cell velocities, and (*E*) spatial correlation function (SCF) of cell velocities were obtained. These three metrics, obtained from the 0- to 5-h time segment, are also shown for convenience. To see this figure in color, go online.

progresses (Fig. 2 *C*). Cells also gradually lost the correlation of velocities over time and over space as indicated by the TCF (Fig. 2 *D*) and SCF (Fig. 2 *E*), respectively. Although cells on a thick Matrigel layer decreased motility in 5–20 h, they continued to move; the 5-h MSD from the three time segments of 5–10, 10–15, and 15–20 h was 9600, 2600, and 2000 μm^2, respectively. This observation reflects the fact that cells in a structure on a thick Matrigel layer continued to move within the structure and also that the entire structure formed by cells underwent a gradual change during this time period.

HeLa cells initially located close to each other later form a bridge between cell aggregates

In time-lapse imaging experiments, we frequently observed that cells initially located close to each other later formed a bridge between cell aggregates. Time-lapse images in Fig. 3 *A* capture a representative case: two cells, 40 μm apart at 0 h, moved in opposite directions in 1 h; one of the two cells (in the green circle) changed its moving direction and started moving toward the other cell (in the cyan circle) at 2.5 h; these two cells finally positioned themselves next to each other at 5 h, bridging the two cell aggregates, one at the bottom-left and the other at the upper-right in the image. The distance between the two cells reflects the movement of the two cells (Fig. 3 *B*). A single cell in our experiments extends up to 400 μm to form a bridge and plays a pivotal role in determining the characteristics of the local structure that cells form (Fig. 3 *C*).

HeLa cells form a large-scale structure in a cell-density-dependent manner

HeLa cells on a thick Matrigel layer started forming a large-scale structure within a half day of cell culture. This structure formation progressed in a cell-density-dependent manner (Fig. 4 *A*). HeLa cells, randomly seeded on a thick Matrigel layer, developed a sparsely distributed structure (referred to as "islands" hereafter) at a low cell density of 200 cells/mm^2 ($N = 3$), a vascular-like structure (referred to as a "network-like" structure hereafter) at an intermediate cell density of 600 cells/mm^2 ($N = 3$), and a large single cluster (referred to as a "continent" hereafter) at a high cell density of 1100 cells/mm^2 ($N = 2$). Additional experiments using HT1080 cells or malignant mesenchymal tumor cells showed that they also formed the three types of structure in a cell-density-dependent manner (see Fig. S1), indicating that the cell-density-dependent structure formation is not only specific to HeLa cells but also common to other types of cancer cell. HeLa cells maintained their structure for at least 1 day (most often for 2–3 days), unlike typical in vitro angiogenesis assays using vascular endothelial cells, in which cells induce apoptosis in 24 h and start losing their

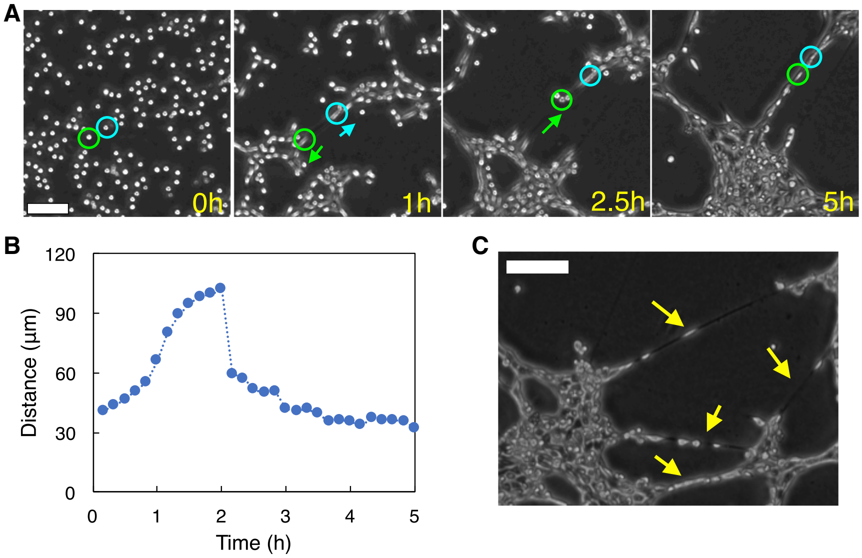

FIGURE 3 Cellular bridge formation. (*A*) Two cells (in *green* and *cyan circles*) that were 40 μm apart at 0 h moved away from each other in 1 h. The cell in the green circle changed its moving direction and started moving toward the cell in the cyan circle at 2.5 h. The two cells formed a bridge between two cell aggregates at 5 h. Scale bar, 100 μm. See Video S5. (*B*) The distance between the two cells in (*A*) versus time. (*C*) Multiple cellular bridges were observed at 18 h. Scale bar, 200 μm. To see this figure in color, go online.

structure (41). Over this long time period, the structure of HeLa cells changed gradually. When a network-like structure was formed, some voids or empty spaces decreased in size and disappeared, whereas other voids increased in size as time progressed (Fig. 4 *A*, network-like).

We analyzed using the two-point correlation function ξ of cell positions the large-scale structures that HeLa cells formed. Note that ξ can exhibit a negative value at some distances. This occurs when the number of cells that exist at such distances is less than that of randomly distributed cells at these distances, indicating that such distances are within voids or emptier spaces (than surrounding spaces). Note also that ξ can exhibit a local minimum at some distances. The distance at which ξ exhibits a local minimum also indicates the length scale of how far cell aggregates are separated from each other. We obtained ξ at 0, 8, 14, 24, and 36 h for the islands, the network-like structure, and the continent that cells formed (Fig. 4 *B*). When the islands were formed, ξ exhibits a local minimum at 370 μm at 24 h and 270 μm at 36 h, respectively, indicating the formation of cell aggregates at these lengths. When the network-like structure was formed, ξ exhibited a local minimum at 690 μm at 24 h and 800 μm at 36 h, indicating that larger aggregates are formed in the network-like structure than in the islands and that the size of aggregates in the network-like structure increased with time. Also, the local

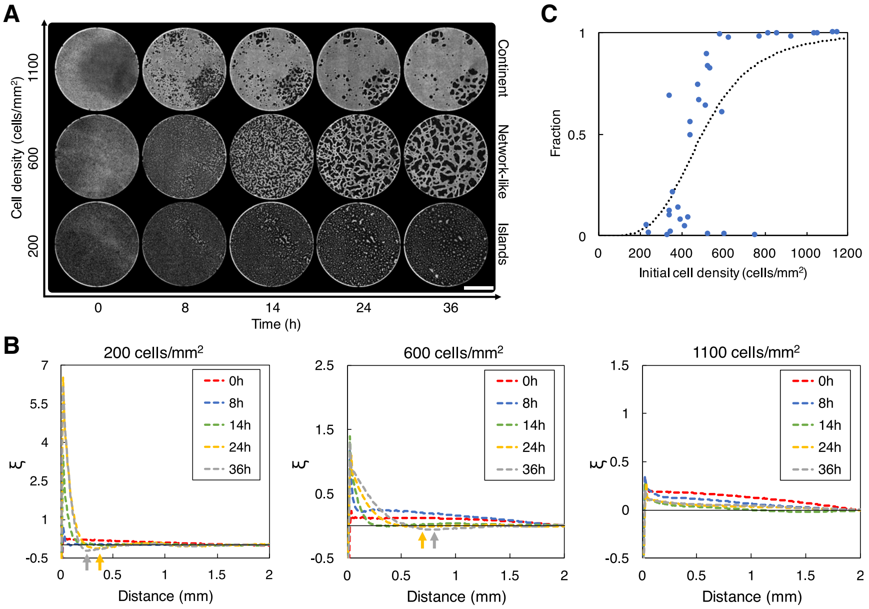

FIGURE 4 Cell-density-dependent structure formation when HeLa cells were plated on a thick Matrigel layer. (*A*) Phase-contrast images of the Matrigel area of 14 mm in diameter. Images were taken at 0, 8, 14, 24, and 36 h after cells were plated. Initial cell densities were 200, 600, and 1100 cells/mm^2. Scale bar, 5 mm. (*B*) Analysis of the large-scale structures that HeLa cells formed on a thick Matrigel layer. The dynamics of the two-point correlation function ξ obtained from the experimental results in (*A*) are shown. Arrows point to local minima in ξ. (*C*) The fraction of cells in the largest cluster at 24 h. Plots represent the experimental results. The dotted curve is obtained by fitting the experimental results to the Hill function. To see this figure in color, go online.

minimum of the network-like structure is not as small as that of the islands, suggesting that aggregates in the network-like structure became interconnected to form a larger structure. When the continent was formed, ξ remained small for all distances, whereas it decreases gradually with distance (Fig. 4 *B*, right), indicating that cells formed a single large aggregate that spans over a long distance (i.e., a continent). Values of ξ also stayed relatively similar at different time instances (Fig. 4 *B*, right), indicating that cells formed a continent at early hours and that the formed continent stayed relatively stable without going through significant changes in its structure.

As the cell density increased, the structure that HeLa cells formed underwent a transition from islands to a network-like structure (Fig. 4 *C*). This transition occurs at a critical cell density ρ_c. To determine ρ_c, we conducted experiments with different initial cell densities and computed the fraction of cells forming the largest cluster at 24 h for each cell density. A cluster is a group of connected cells, and two cells are considered connected when their separation distance is smaller than 50 μm. We used the Hill function in the form of $(\rho^q/(\rho^q + \rho_c^q))$, where q is the Hill coefficient and ρ is the cell density, to fit the experimental results (dotted line in Fig. 4 *C*) and obtained $q = 4.0$ and $\rho_c = 498$ cells/μm^2.

Roles of remote and contact forces in large-scale multicellular structure formation

A key to understanding how cells interact and form a large-scale structure is to identify major forces that act between cells and develop a simple model based on such forces. In this work, we considered two types of force: remote and contact forces. The remote force attracts cells at a distance toward each other. This force exponentially decreases with the distance between cells (as seen in Eq. 7). The contact force attracts cells in physical contact toward each other. This force linearly increases with the distance between cells that are in physical contact (as seen in Eq. 8). Fig. 5 *A* shows the remote and contact forces that a cell receives from another cell as a function of their separation distance, respectively.

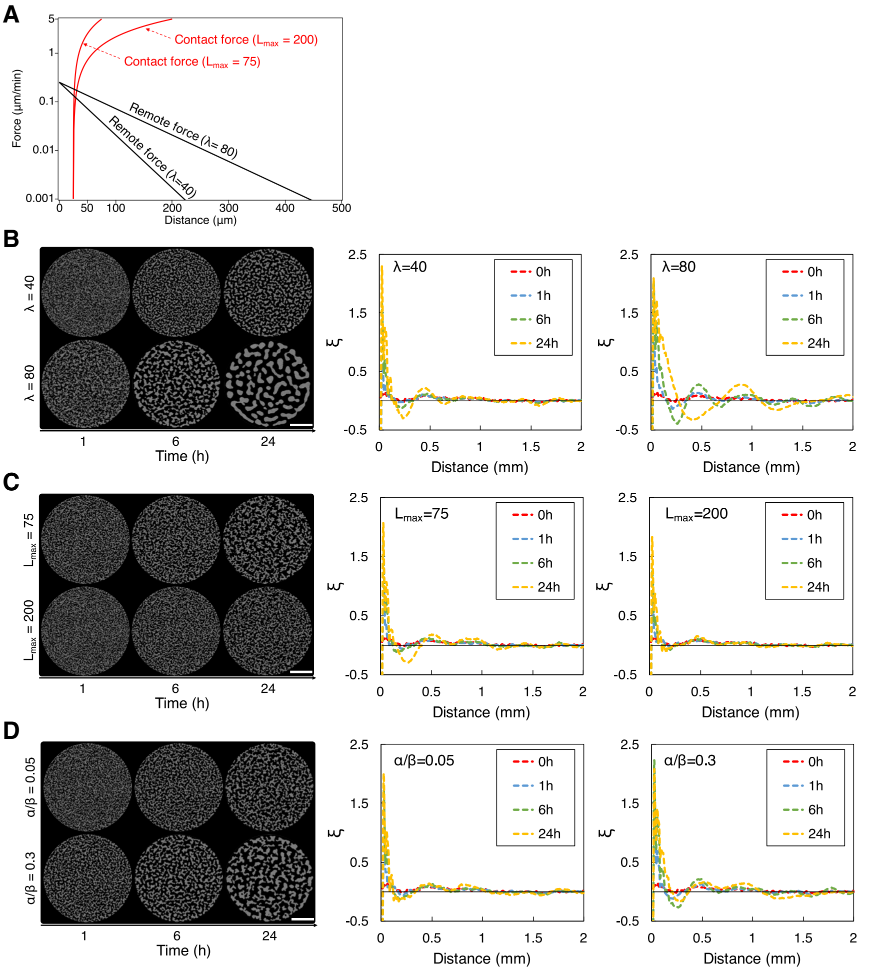

FIGURE 5 Simulation results. (*A*) The remote and contact forces that a cell receives from another cell as a function of cell-cell distance. The vertical axis is in logarithmic scale. $\alpha = 0.25$ μm/min, $\lambda = 40$ or 80 μm, $\beta = 5$ μm/min, and $L_{max} = 75$ or 200 μm. These values are used in (*B*)–(*D*) unless otherwise noted. (*B–D*) Evolution of the structure that cells formed and two-point correlation function ξ of the formed structure when only the remote force was enabled, when only the contact force was enabled, and when both forces were enabled ($(\alpha, \beta) = (0.25, 5)$ or $(1.5, 5)$), respectively. Scale bars, 2 mm. In (*B*)–(*D*) left, cells are drawn as a circle of $L_{min} = 25$ μm in diameter. The simulated area of $2R = 8$ mm in diameter is shown. In (*B*)–(*D*) center and right, the solid (*black*) line represents $\xi = 0$. To see this figure in color, go online.

Using simulations, we examined how cells use the remote and contact forces to form a large-scale structure. We considered the following three cases. In the first case, we enabled the remote force and disabled the contact force. In the second case, we disabled the remote force and enabled the contact force. In the third case, we enabled both forces.

In the first case, in which only the remote force was enabled, α and λ in Eq. 7 are the key parameters; α represents the strength of the remote force and determines how quickly cells move and form a structure (i.e., the timescale of the structure), and λ is the characteristic length of the remote force and determines the spatial characteristics of the structure that cells form (i.e., the spatial scale of the structure), provided that the volume exclusion effect in Eq. 9 is negligibly small and only effective in a small distance range. In this first case, we set $\alpha = 0.25$ μm/min, such that cells form a relatively stable structure within 24 h to match the timescale of the structure formation observed in experiments, whereas we varied the value of λ to examine the spatial characteristics of the structure. When $\lambda = 40$ μm, cells first formed small aggregates, and such small aggregates further aggregated and formed larger aggregates and edges (i.e., multiple small aggregates aligned side by side) (Fig. 5 *B*, left). This process repeated to form a number of disconnected islands at 24 h. When λ increased to 80 μm, cells formed larger aggregates at 1 and 6 h and eventually a coarser island structure at 24 h than when $\lambda = 40$ μm (Fig. 5 *B*, left). The two-point correlation function ξ clearly captures the formation of cell aggregates of different sizes between when $\lambda = 40$ (Fig. 5 *B*, center) and when $\lambda = 80$ (Fig. 5 *B*, right); at 24 h, ξ exhibits a local minimum at 230 μm when $\lambda = 40$ μm and at 400 μm when $\lambda = 80$ μm, showing that at these distances, a smaller number of cells exist than when cells are randomly distributed and that voids or emptier spaces are formed at around these distances. These results show that, when λ becomes larger or when the remote force travels farther, cells form a coarser structure.

In the second case, in which only the contact force was enabled, β and $L_{\max}$ in Eq. 8 are the key parameters; similarly to α in the first case, β represents the strength of the contact force and determines the timescale of the structure, and $L_{\max}$ is the contact termination distance and determines the spatial scale of the structure. In this second case, we set $\beta = 5$ μm/min, such that cells form a relatively stable structure within 24 h, whereas we varied the value of $L_{\max}$ to examine the spatial characteristics of the structure. When $L_{\max} = 75$ μm, cells first formed small aggregates and narrow edges (Fig. 5 *C*, left), and such small aggregates and narrow edges further developed to become interconnected. This process resulted in a large-scale structure at 24 h consisting of substructures that are complex in shape (e.g., irregular and asymmetric shapes combining multiple voids and multiple needle-like narrow edges within aggregates) (Fig. 5 *C*, left). This large-scale structure is spatially distinct from the disconnected islands in the first case. This structure reflects the characteristics of the contact force; it decreases as the cells move closer to each other, allowing cells to remain distributed; it also travels only through cell-cell contact, allowing cells to form needle-like narrow edges. When $L_{\max}$ increased to 200 μm, cells formed smaller aggregates and narrower edges at 1 and 6 h than when $L_{\max} = 75$ (Fig. 5 *C*, left). We note that this seemingly counterintuitive behavior is due to how we varied the parameters in simulations; we kept β constant and varied $L_{\max}$. The contact force at a given cell-cell distance, thus, became weaker and aggregated cells at a slower speed when $L_{\max}$ is larger (Fig. 5 *A*). As a result, the contact force with larger $L_{\max}$ requires more time to form aggregates and, when observed before sufficient time passes in simulations, forms smaller cell aggregates than with smaller $L_{\max}$. The two-point correlation function ξ exhibits a local minimum at 270 μm at 24 h, when $L_{\max} = 75$ μm (Fig. 5 *C*, center), clearly indicating the formation of voids at around this distance. On the contrary, when $L_{\max} = 200$ μm, ξ gradually decreases with distance (Fig. 5 *C*, right) and does not exhibit an apparent local minimum at 24 h, indicating that most cells and cell aggregates are not completely isolated from each other. These results show that, when $L_{\max}$ becomes larger or when the contact force travels farther, cells form a finer and less coarse structure.

In the third case, we enabled both remote and contact forces. In this case, we first set $\alpha = 0.25$ μm/min and $\lambda = 40$ μm (same as those in the first case) and $\beta = 5$ μm/min and $L_{\max} = 200$ μm (same as those in the second case). In this case (Fig. 5 *D*, left, $\alpha/\beta = 0.05$), cells formed a structure with characteristics that are between those observed in the first case with $\lambda = 40$ (Fig. 5 *B*, left) and the second case with $L_{\max} = 200$ (Fig. 5 *C*, left); cell aggregates were more connected to each other and more complex in shape than in the first case ($\lambda = 40$) and less connected to each other and simpler in shape than in the second case ($L_{\max} = 200$). The two-point correlation function clearly captures this characteristic of the structure (Fig. 5 *D*, center); the distance at which ξ takes a local minimum at 24 h in this third case lies between the distances at which ξ takes minima in the first and second cases. When the remote force increased its strength from $\alpha = 0.25$ to 1.5 μm/min while keeping $\beta = 5$ μm/min (Fig. 5 *D*, left, $\alpha/\beta = 0.3$), cells formed at 24 h a structure that consists of larger substructures than those with $\alpha/\beta = 0.05$. This demonstrates that, when the remote force is stronger, it attracts cells at a faster speed, and cell aggregates grow in size faster. The two-point correlation function ξ captures this characteristic of the structure. When $\alpha/\beta = 0.3$, ξ at 24 h exhibits a smaller local minimum at long distances (for instance, at the distance of 1.25 mm) than when $\alpha/\beta = 0.05$ (Fig. 5 *D*, center and right), indicating that larger cell aggregates are formed with $\alpha/\beta = 0.3$ than

with $\alpha/\beta = 0.05$. These results show how the ratio of α/β controls the distribution of cells within the structure formed.

Roles of remote and contact forces in the determination of the critical cell density

We examined through simulations the roles of remote and contact forces in determining the critical cell density ρ_c. As observed in experiments (Fig. 4), different initial cell densities result in cells forming different types of large-scale structure (islands, a network-like structure, and a continent). As with the critical cell density in experiments, we define ρ_c as the initial cell density at which the resulting structure of cells transits from a group of disconnected cell aggregates (i.e., islands) to a connected structure of all cells (i.e., either a network-like structure or a continent). We considered the three cases (remote force only, contact force only, and both forces enabled) as in the previous simulations and varied the initial cell density ρ from 200 to 1000 cells/mm^2. By comparing the structure that cells formed at 24 h in simulations (Fig. 6 *A*), we found that, in all three cases, the critical cell density ρ_c exists, and the structure of cells changes from islands to a connected structure of all cells around ρ_c. The value of ρ_c differs in the three cases (Fig. 6 *B*): $\rho_c = 820$ cells/mm^2 when only the remote force is enabled, $\rho_c = 570$ cells/mm^2 when only the contact force is enabled, and $\rho_c = 630$ cells/mm^2 when both forces are enabled. This reflects the different characteristics of the two forces. The remote force allows nearby cells to quickly aggregate and form isolated islands, requiring a higher cell density to form a fully connected structure, whereas the contact force allows cells to remain distributed and form a connected structure at a lower cell density. Note that the values of ρ_c depend on key parameters of the remote and contact forces, such as λ, $L_{\max}$, and α/β, reflecting the characteristics of the two forces (see Fig. S2). Note further that, in addition to the critical cell density, the critical value also exists for key parameters of the remote and contact forces, such as λ, $L_{\max}$, and α/β (see Fig. S3). At the critical value of these parameters, the fraction of the cells in the largest cluster becomes 0.5, and the structure of cells transits to a different type of structure at the critical value of such parameters.

Reproducing the cellular bridge formation process

We observed through time-lapse imaging that cells initially located close to each other played a key role in forming a bridge between cell aggregates (Fig. 3). We speculate that this is because a cell made physical contact with some nearby cells in early phases of the structure formation, maintained some such physical contacts while it moved, and became interconnected cell aggregates. Fig. 7 *A* reproduces through simulations the experimental observation made in Fig. 3 *A*. At 0 h, cells in Fig. 7 *A* were distributed according to the experimentally observed cell distribution in Fig. 3 *A*. The green and cyan cells were initially within the contact initiation distance and thus formed a physical contact. At 1 h, the two cells moved in the opposite direction. This opposite movement of the two cells is because the sum of the remote force and the contact force that the green cell receives and that the cyan cell receives were pointing to the opposite directions. As the two cells moved to the opposite direction, the "link" between the cells stretched, implying that these cells elongated. The link between the two cells then bridged the two cell aggregates, one at the bottom-left and the other at the upper-right in the image.

The experimental results in Fig. 3 *A* show that the green cell changed its moving direction and started moving toward the cyan cell at 2.5 h. We speculate that this sudden reversal of cell movement occurred because the green cell lost its physical contacts with some nearby cells or the cell moved preferentially toward the cyan cell through the contact with the cyan cell. In simulations, at 2.5 h, we artificially disabled all physical contacts that the green cell maintained, except for one with the cyan cell. The green cell then moved toward the cyan cell at 2.5 h because of the contact force it received from the cyan cell. At 5 h, the green cell moved close to the cyan cell, as observed in Fig. 3 *A*, bridging the two cell

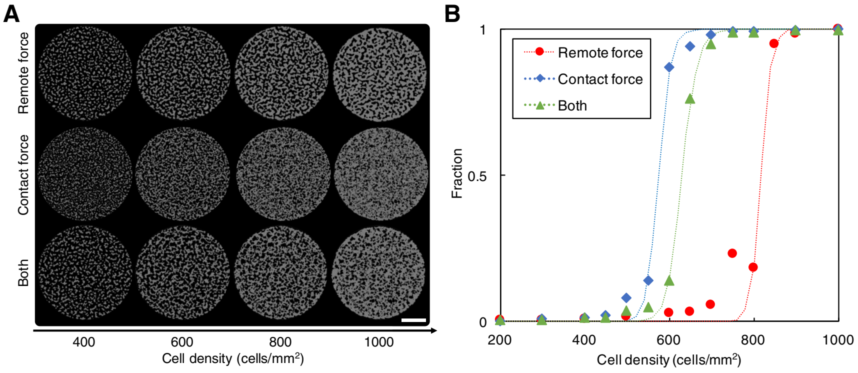

FIGURE 6 Impact of the cell density. (*A*) Structures formed at 24 h at cell densities of 400, 600, 800, and 1000 cells/mm^2 when the remote force is enabled ($\alpha = 0.25$ μm/min and $\lambda = 40$ μm), when the contact force is enabled ($\beta = 5$ μm/min and $L_{\max} = 200$ μm), and when both forces are enabled ($\alpha = 0.25$ μm/min, $\beta = 5$ μm/min, $\lambda = 40$ μm, and $L_{\max} = 200$ μm). Simulated area of $2R = 8$ mm in diameter is shown. Scale bar, 2 mm. (*B*) The fractions of cells in the largest cluster in the three cases in (*A*). Symbols represent simulation results. Dotted curves are obtained by fitting the simulation results to the Hill function. To see this figure in color, go online.

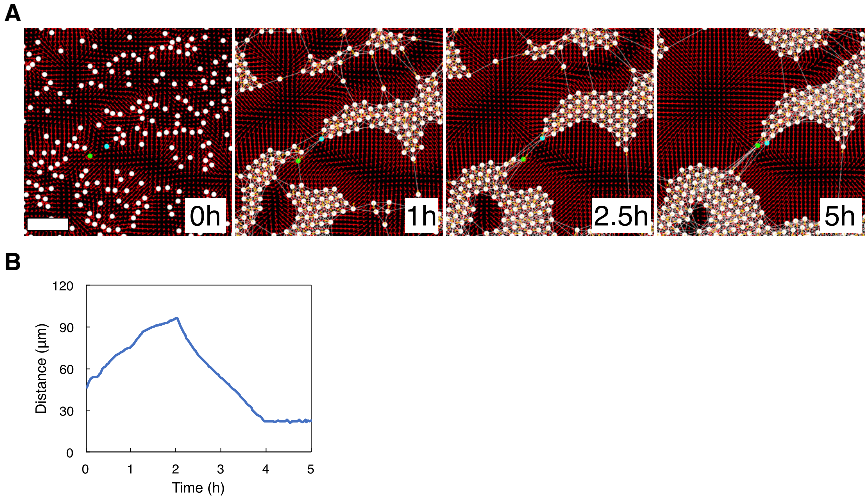

FIGURE 7 (*A*) Cellular bridge formation in simulations. White circles represent cells, and links between circles represent physical contacts between cells. Green and cyan circles correspond to the two cells observed in Fig. 3 *A*. The red arrow at a position represents the remote force generated by all cells for the position in the simulated area. The yellow arrow at a cell represents the contact force that the cell receives from all cells with which the cell is in physical contact. Each arrow points to the direction of the force, and the length of the arrow indicates the strength of the force. Scale bar, 100 μm. See Video S6. (*B*) The distance between the green and cyan cells versus time. The following parameter values were used in simulations: $L_{\min} = 25$ μm, $\alpha = 0.45$ μm/min, $\lambda = 49$ μm, $\beta = 10$ μm/min, and $L_{\max} = 200$ μm. Default values were used for other parameters. To see this figure in color, go online.

aggregates. The distance between the green and cyan cells in simulations well reproduces that in experiments (Fig. 3 *B*).

Reproducing the large-scale multicellular structure formation process

We examined whether our simple model reproduces the large-scale multicellular structures observed in experiments. We obtained the initial cell positions from the experiments at 0 h (Fig. 4 *A*) and used them in simulations. Simulation results (Fig. 8 *A*) showed that cells form the three types of structure in a cell-density-dependent manner, as we observed in experiments (Fig. 4 *A*): the islands at a low cell density, the network-like structure at an intermediate cell density, and the continent at a high cell density. The two-point correlation function ξ confirmed that the structures formed in simulations (Fig. 8 *B*) have similar characteristics as those found in experiments (Fig. 4 *B*).

The simulation results showed that the large-scale multicellular structure significantly depends on the initial cell distribution or the fluctuations in the initial cell distribution (see Fig. S4). When the nonrandom initial cell distribution from experiments was used in simulations, cells formed a network-like structure consisting of both isolated aggregates and interconnected aggregates of different shapes and sizes (Fig. S4 *A*, experiment); some are small and isolated, like those seen in the island structure, and some are very large and connected smaller aggregates similarly to those that comprise the continent structure. Fluctuations in the initial cell distribution served as seeds for cells to form these diverse aggregates of different shapes and sizes. These diverse aggregates of different shapes and sizes that cells formed in a network-like structure in simulations resemble those in the network-like structure that HeLa cells formed in experiments (Fig. 4 *A*, network-like).

We also examined through simulations how a large-scale multicellular structure changes over an extended period of time. Simulation results showed that, over one to four simulated days, the network-like structure gradually changes its structure and becomes coarser, while some edges become wider, some aggregates and voids become larger, and some (small) voids disappear (Fig. 8 *C*). These changes observed in simulations are consistent with the experimental observations (Fig. 4 *A*, network-like) that the network-like structure changed gradually.

DISCUSSION

In this work, we observed that HeLa cells move aggressively on Matrigel and that HeLa cell motility depends on the Matrigel thickness. The increased motility of HeLa cells could have arisen from their reduced adhesion to the Matrigel surface, as cell motility and cell adhesion to the surface are in general inversely correlated (29). In addition to the reduced adhesion to the surface, additional factors could have contributed to increased cell motility because the 5-h MSD of HeLa cells on a thick Matrigel layer was a magnitude larger than that on a thin Matrigel layer, and HeLa cells on a thick Matrigel layer coordinated their movement at the length scale of up to ~1 mm, whereas cells on a thin Matrigel layer exhibited no coordinated motion at that length scale. We speculate that HeLa cells interact with Matrigel, generate a remote force that propagates over the Matrigel, and coordinate their movements with other cells at distance. We further speculate that the Matrigel thickness determines the strength of remote force and the distance over which the remote force travels over the Matrigel.

We also observed through time-lapse imaging that HeLa cells that were initially located close to each other later formed a bridge and connected two cell aggregates. This is consistent with epithelial bridges reported in (21,22), in which human bronchial epithelial cells formed bridges hundreds of microns long and facilitated cell migration between the bridged cell clusters. We speculate that some HeLa cells

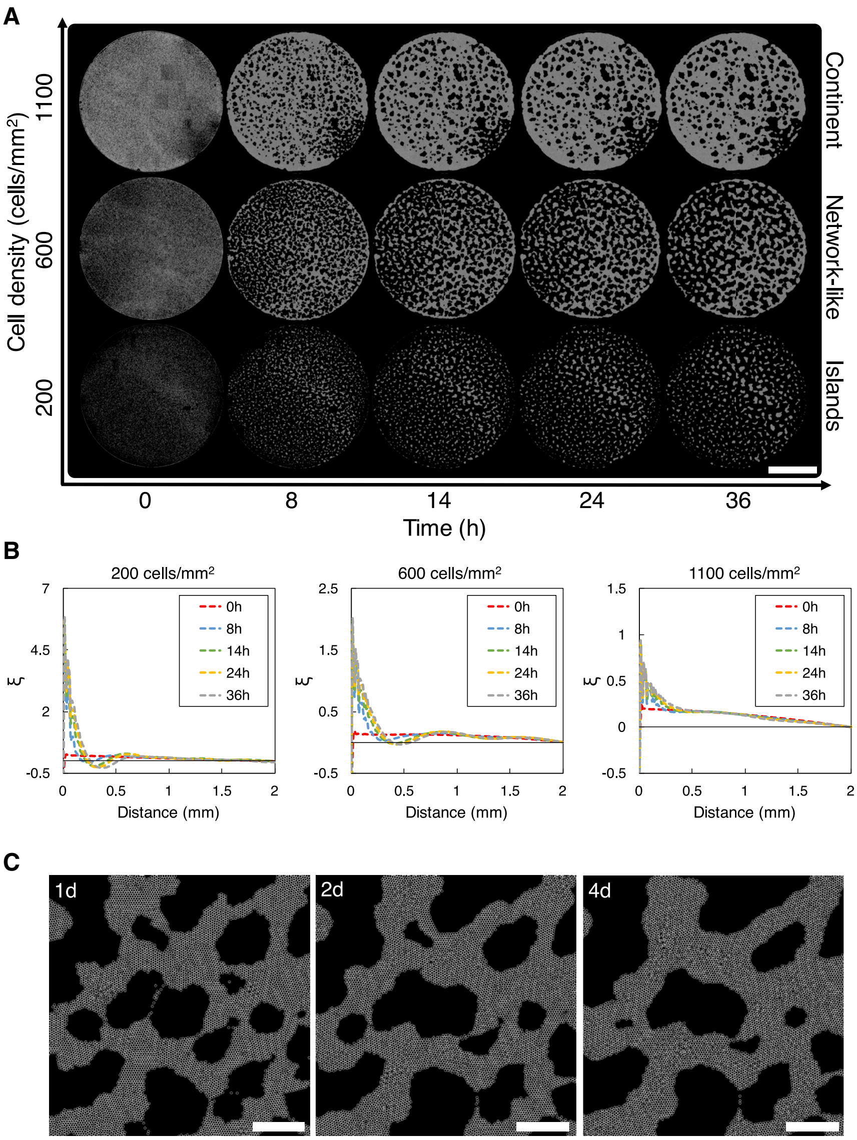

FIGURE 8 Simulation results reproduce the spatiotemporal structures of cells observed in experiments (Fig. 4 *A*). (*A*) The structures of cells in simulations at 0, 8, 14, 24, and 36 h. $\alpha = 0.25$ μm/min, $\lambda = 80$ μm, $\beta = 5$ μm/min, and $L_{\max} = 200$ μm. The initial cell distribution and the cell density of approximately ρ = 200, 600, and 1100 cells/mm^2 are from the experiments. The simulated area of $2R = 14$ mm in diameter is shown. Scale bar, 5 mm. See Videos S7–S9. (*B*) The two-point correlation function ξ obtained from simulations at 0, 8, 14, 24, and 36 h. (*C*) Long-term behavior of a multicellular structure formed in simulations. The simulation in (*A*) (network-like) was run over a time period of 4 days. A part of the simulated area was shown. Scale bars, 1 mm. To see this figure in color, go online.

made physical contacts with other nearby HeLa cells in early phases of our experiments, maintained their physical contact while moving independently, and at a later time, formed a bridge. This suggests that the contact force contributes to the formation of cellular bridges and, consequently, to the formation of a large-scale multicellular structure.

In our experiments, HeLa cells formed a network-like structure on a thick Matrigel layer in a cell-density-dependent manner. The process through which HeLa cells formed a network-like structure resembles, for the most part, that of typical in vitro angiogenesis assays using vascular endothelial cells; cells attach to the matrix surface in 1 h and migrate toward each other over the next 2–4 h; they then form capillary-like cellular bridges, which mature by 6–16 h; after 24 h, cells undergo apoptosis, and bridges detach from the matrix and break apart (9,41,42). We, however, note that HeLa cells formed more stable structures and maintained their structures for a longer time period of 2–3 days and that they gradually developed wider cellular bridges than vascular endothelial cells. These differences demonstrate the intrinsic nature of HeLa cells to form an epithelial sheet.

We presented a simple model to understand through simulations how HeLa cells form a large-scale structure. The model considers two types of cell-cell attraction force that we observed through experiments: the remote force, which is exerted by one cell on another at a distance, and the contact force, which acts between two cells in physical contact. The remote force represents chemotactic (9,34,43), haptotactic (12,29), and mechanotactic forces (14–18), whereas the contact force represents cadherin-dependent cell-cell attraction force (19,20) and epithelial bridges (21,22). Our model is deterministic and includes the minimal number of parameters, representing the simplest among

the vascular-like structure formation models known in the literature (9,14,16,30,34,43,44).

Simulations using the simple model verified various observations made in experiments. The model reproduces the cellular bridge observed in the experiment formation process, predicts the existence of critical parameter values, and reproduces statistical characteristics (i.e., two-point correlation function of cell positions) of the experimentally observed structures of HeLa cells. We also note that the model accounts for the material of the substrate (i.e., a thin Matrigel layer or a glass surface) to reproduce cell motility observed in early phases of experiments (see Fig. S5 and its accompanying text).

Simulation results suggest that the remote and contact forces in the simple model are dominant factors and determine the large-scale structure of HeLa cells observed in experiments. Simulation results also suggest that other factors contributing to the formation of a large-scale multicellular structure may be expressed in the form of either the remote force, the contact force, or the combination of both. Experiments should verify these findings obtained through simulations and quantify how the remote and contact forces help cells form a large-scale multicellular structure. For instance, one may experimentally identify the underlying physical mechanisms that induce the remote force and/or the contact force, vary key parameters of such mechanisms, examine whether the critical cell density shifts in a manner predicted by simulations, and quantify the degree to which the remote and contact forces contribute to forming a large-scale structure. Such experiments await future research.

It is desirable to experimentally identify the underlining physical mechanisms that induce the remote and contact forces and to extend our model to include details of the identified underlying mechanisms. A promising direction is to experimentally investigate whether the Matrigel and the deformation that moving cells create on the Matrigel are among the underlying physical mechanisms of the remote force. We frequently observed in experiments that Matrigel deforms as cells move and that such deformation is sometimes permanent (data not shown). In addition, cells constantly secrete and degrade ECM proteins to participate in ECM remodeling (45). When experiments verify that the Matrigel and its deformation are among the underlying physical mechanisms of the remote force, our model can be extended to describe the dynamics of Matrigel and the effect of the Matrigel on cell motion (15,36,44,46).

Our experimental and simulation results provide important implications for cancer cell biology. We demonstrated how key parameters such as the initial cell density and force parameters affect the fraction of cells that belongs to the largest cluster. This suggests that, by manipulating these parameters, one can prevent cancer cells from forming a network-like structure or vasculogenic mimicry (1–5). This is important because cancer cells form vasculogenic mimicry to gain access to blood vessels and nutrient sources cooperatively to sustain their life.

SUPPORTING MATERIAL

Supporting Material can be found online at https://doi.org/10.1016/j.bpj.2020.01.037.

AUTHOR CONTRIBUTIONS

T.N., T.S., Y.H., and T.H. designed the research. T.N. and Y.K. performed biological experiments under the guidance of Y.H. and T.H. T.N., Y.O., and T.S. developed mathematical models. T.N. and Y.O. performed computer simulations and data analysis. T.N. and T.S. wrote the article.

ACKNOWLEDGMENTS

This work was supported by JSPS KAKENHI grants: JP17H00733 to T.N.; JP17H01444 and JP18H05533 to Y.H.; JP17H03636 and JP18H05528 to T.H.

REFERENCES

1. Folberg, R., M. J. Hendrix, and A. J. Maniotis. 2000. Vasculogenic mimicry and tumor angiogenesis. *Am. J. Pathol.* 156:361–381.
2. Hendrix, M. J. C., E. A. Seftor, …, R. E. B. Seftor. 2003. Vasculogenic mimicry and tumour-cell plasticity: lessons from melanoma. *Nat. Rev. Cancer.* 3:411–421.
3. Wan, H.-Y., Q.-Q. Li, …, H. Tang. 2014. MiR-124 represses vasculogenic mimicry and cell motility by targeting amotL1 in cervical cancer cells. *Cancer Lett.* 355:148–158.
4. Williamson, S. C., R. L. Metcalf, …, C. Dive. 2016. Vasculogenic mimicry in small cell lung cancer. *Nat. Commun.* 7:13322.
5. Racordon, D., A. Valdivia, …, G. I. Owen. 2017. Structural and functional identification of vasculogenic mimicry in vitro. *Sci. Rep.* 7:6985.
6. Budrene, E. O., and H. C. Berg. 1991. Complex patterns formed by motile cells of *Escherichia coli*. *Nature.* 349:630–633.
7. Shapiro, J. A. 1995. The significances of bacterial colony patterns. *BioEssays.* 17:597–607.
8. Guillot, C., and T. Lecuit. 2013. Mechanics of epithelial tissue homeostasis and morphogenesis. *Science.* 340:1185–1189.
9. Serini, G., D. Ambrosi, …, F. Bussolino. 2003. Modeling the early stages of vascular network assembly. *EMBO J.* 22:1771–1779.
10. Calderon, G. A., P. Thai, …, J. S. Miller. 2017. Tubulogenesis of co-cultured human iPS-derived endothelial cells and human mesenchymal stem cells in fibrin and gelatin methacrylate gels. *Biomater. Sci.* 5:1652–1660.
11. Fessel, A., C. Oettmeier, …, H.-G. Döbereiner. 2012. *Physarum polycephalum* percolation as a paradigm for topological phase transitions in transportation networks. *Phys. Rev. Lett.* 109:078103.
12. Smith, J. T., J. K. Tomfohr, …, W. M. Reichert. 2004. Measurement of cell migration on surface-bound fibronectin gradients. *Langmuir.* 20:8279–8286.
13. Yin, Z., D. Noren, …, A. Levchenko. 2008. Analysis of pairwise cell interactions using an integrated dielectrophoretic-microfluidic system. *Mol. Syst. Biol.* 4:232.
14. Manoussaki, D., S. R. Lubkin, …, J. D. Murray. 1996. A mechanical model for the formation of vascular networks *in vitro*. *Acta Biotheor.* 44:271–282.

15. Murray, J. D. 2003. On the mechanochemical theory of biological pattern formation with application to vasculogenesis. *C. R. Biol.* 326:239–252.
16. van Oers, R. F. M., E. G. Rens, …, R. M. H. Merks. 2014. Mechanical cell-matrix feedback explains pairwise and collective endothelial cell behavior in vitro. *PLoS Comput. Biol.* 10:e1003774.
17. Reinhart-King, C. A., M. Dembo, and D. A. Hammer. 2008. Cell-cell mechanical communication through compliant substrates. *Biophys. J.* 95:6044–6051.
18. Rørth, P. 2009. Collective cell migration. *Annu. Rev. Cell Dev. Biol.* 25:407–429.
19. Hayer, A., L. Shao, …, T. Meyer. 2016. Engulfed cadherin fingers are polarized junctional structures between collectively migrating endothelial cells. *Nat. Cell Biol.* 18:1311–1323.
20. Ladoux, B., and R.-M. Mège. 2017. Mechanobiology of collective cell behaviours. *Nat. Rev. Mol. Cell Biol.* 18:743–757.
21. Zani, B. G., and E. R. Edelman. 2010. Cellular bridges: routes for intercellular communication and cell migration. *Commun. Integr. Biol.* 3:215–220.
22. Zani, B. G., L. Indolfi, and E. R. Edelman. 2010. Tubular bridges for bronchial epithelial cell migration and communication. *PLoS One.* 5:e8930.
23. Kleinman, H. K., and G. R. Martin. 2005. Matrigel: basement membrane matrix with biological activity. *Semin. Cancer Biol.* 15:378–386.
24. Meijering, E., O. Dzyubachyk, and I. Smal. 2012. Methods for cell and particle tracking. *Methods Enzymol.* 504:183–200.
25. Eisenstein, D. J., I. Zehavi, …, J. A. Frieman. 2005. Detection of the baryon acoustic peak in the large-scale correlation function of SDSS luminous red galaxies. *Astrophys. J.* 633:560–574.
26. Grima, R. 2008. Multiscale modeling of biological pattern formation. *Curr. Top. Dev. Biol.* 81:435–460.
27. Perfahl, H., B. D. Hughes, …, H. M. Byrne. 2017. 3D hybrid modelling of vascular network formation. *J. Theor. Biol.* 414:254–268.
28. Stokes, C. L., D. A. Lauffenburger, and S. K. Williams. 1991. Migration of individual microvessel endothelial cells: stochastic model and parameter measurement. *J. Cell Sci.* 99:419–430.
29. Smith, J. T., J. T. Elkin, and W. M. Reichert. 2006. Directed cell migration on fibronectin gradients: effect of gradient slope. *Exp. Cell Res.* 312:2424–2432.
30. Szabo, A., E. D. Perryn, and A. Czirok. 2007. Network formation of tissue cells via preferential attraction to elongated structures. *Phys. Rev. Lett.* 98:038102.
31. Sepúlveda, N., L. Petitjean, …, V. Hakim. 2013. Collective cell motion in an epithelial sheet can be quantitatively described by a stochastic interacting particle model. *PLoS Comput. Biol.* 9:e1002944.
32. Middleton, A. M., C. Fleck, and R. Grima. 2014. A continuum approximation to an off-lattice individual-cell based model of cell migration and adhesion. *J. Theor. Biol.* 359:220–232.
33. Camley, B. A., J. Zimmermann, …, W.-J. Rappel. 2016. Emergent collective chemotaxis without single-cell gradient sensing. *Phys. Rev. Lett.* 116:098101.
34. Gamba, A., D. Ambrosi, …, F. Bussolino. 2003. Percolation, morphogenesis, and burgers dynamics in blood vessels formation. *Phys. Rev. Lett.* 90:118101.
35. Wartlick, O., A. Kicheva, and M. González-Gaitán. 2009. Morphogen gradient formation. *Cold Spring Harb. Perspect. Biol.* 1:a001255.
36. Czirok, A. 2013. Endothelial cell motility, coordination and pattern formation during vasculogenesis. *Wiley Interdiscip. Rev. Syst. Biol. Med.* 5:587–602.
37. Daub, J. T., and R. M. Merks. 2013. A cell-based model of extracellular-matrix-guided endothelial cell migration during angiogenesis. *Bull. Math. Biol.* 75:1377–1399.
38. Wei, G., P. Bogdan, and R. Marculescu. 2013. Bumpy rides: modeling the dynamics of chemotactic interacting bacteria. *IEEE J. Sel. Areas Comm.* 31:879–890.
39. Koorehdavoudi, H., P. Bogdan, …, M. Sitti. 2017. Multi-fractal characterization of bacterial swimming dynamics: a case study on real and simulated Serratia marcescens. *Proc. Math. Phys. Eng. Sci.* 473:20170154.
40. Allen, M. P. 2004. Introduction to molecular dynamics simulation. *In* Computational Soft Matter: From Synthetic Polymers to Proteins. N. Attig, K. Binder, H. Grubmuller, and K. Kremer, eds. John von Neumann Institute for Computing, pp. 1–28.
41. Arnaoutova, I., and H. K. Kleinman. 2010. In vitro angiogenesis: endothelial cell tube formation on gelled basement membrane extract. *Nat. Protoc.* 5:628–635.
42. Khoo, C. P., K. Micklem, and S. M. Watt. 2011. A comparison of methods for quantifying angiogenesis in the Matrigel assay *in vitro*. *Tissue Eng. Part C Methods.* 17:895–906.
43. Merks, R. M., S. V. Brodsky, …, J. A. Glazier. 2006. Cell elongation is key to in silico replication of in vitro vasculogenesis and subsequent remodeling. *Dev. Biol.* 289:44–54.
44. Namy, P., J. Ohayon, and P. Tracqui. 2004. Critical conditions for pattern formation and in vitro tubulogenesis driven by cellular traction fields. *J. Theor. Biol.* 227:103–120.
45. Lu, P., V. M. Weaver, and Z. Werb. 2012. The extracellular matrix: a dynamic niche in cancer progression. *J. Cell Biol.* 196:395–406.
46. Vernon, R. B., J. C. Angello, …, E. H. Sage. 1992. Reorganization of basement membrane matrices by cellular traction promotes the formation of cellular networks in vitro. *Lab. Invest.* 66:536–547.

Biophysical Journal, Volume *118*

Supplemental Information

Roles of Remote and Contact Forces in Epithelial Cell Structure Formation

Tadashi Nakano, Yutaka Okaie, Yasuha Kinugasa, Takako Koujin, Tatsuya Suda, Yasushi Hiraoka, and Tokuko Haraguchi

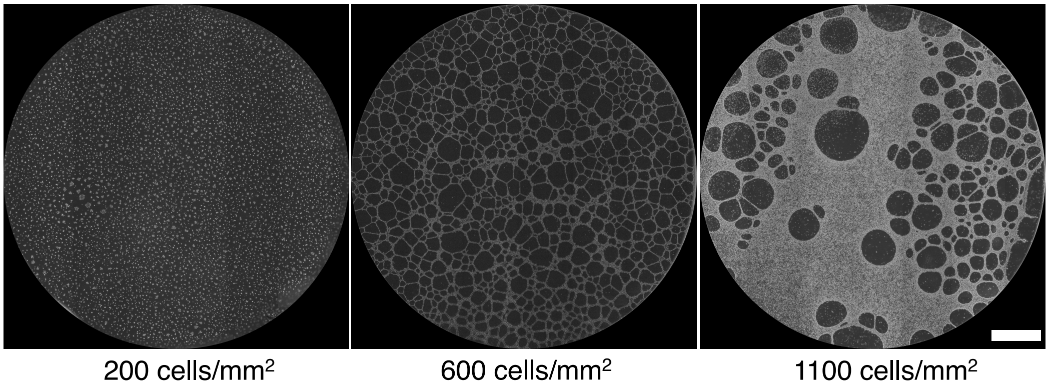

Figure S1. Cell-density dependent structure formation of HT1080 cells (malignant mesenchymal tumor cells) in experiments. Cells were plated on a thick Matrigel layer, and phase-contrast images of the Matrigel area of 14 mm of diameter were taken at 24 h after cells were plated. Similarly to HeLa cells (Fig. 4), HT1080 cells formed islands when the cell density is 200 cells/mm^2 (N = 3), a network-like structure when the cell density is 600 cells/mm^2 (N = 3), and a continent when the cell density is 1100 cells/mm^2 (N = 1). Scale bar, 2 mm.

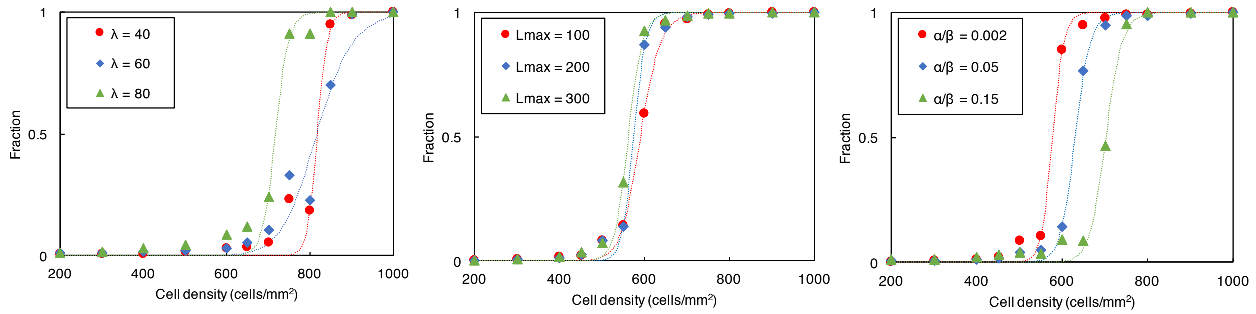

Figure S2. Simulation results on the fraction of cells in the largest cluster as a function of cell density, *(left)* when only the remote force is enabled ($\alpha = 0.25$ μm/min), *(center)* when only the contact force is enabled ($\beta = 5$ μm/min), and *(right)* when both forces are enabled (α is varied, $\beta = 5$ μm/min, $\lambda = 40$ μm, $L_{\max} = 200$ μm), respectively. The simulated area is $2R = 8$ mm. The structure of cells formed at 24 h is examined. Plots represent simulation results, and dotted curves are obtained by fitting the simulation results to the Hill function. The critical cell densities are *(left)* 820, 820, and 720 cells/mm^2 when $\lambda = 40$, 60, and 80 μm, *(center)* 590, 570, and 560 cells/mm^2 when $L_{\max} = 100$, 200, and 300 μm, *(right)* 580, 630, and 700 cells/mm^2 when $\alpha/\beta = 0.002$, 0.05, and 0.15, respectively.

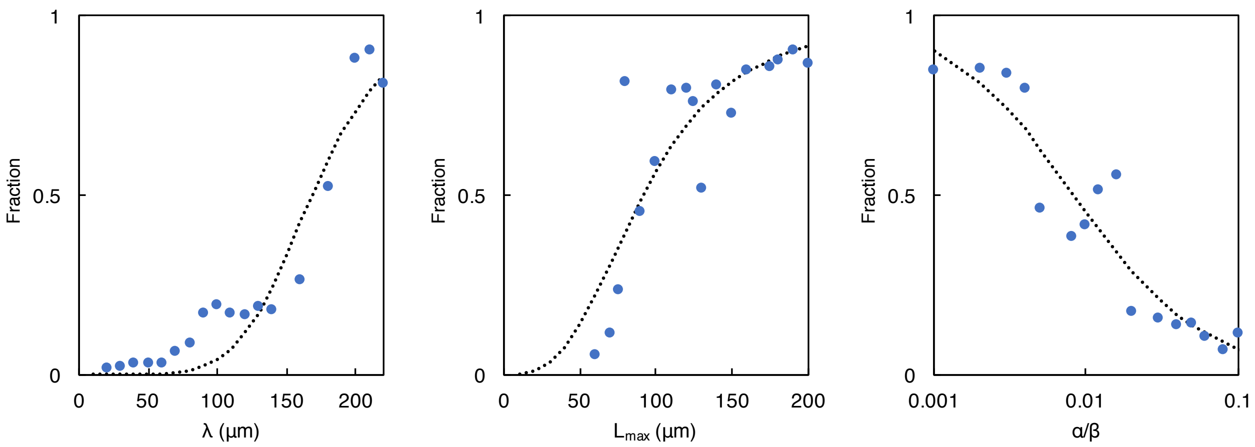

Figure S3. Simulation results on the fraction of cells in the largest cluster *(left)* as a function of λ when only the remote force is enabled (α = 0.25 μm/min), *(center)* as a function of L_{max} when only the contact force is enabled (β = 5 μm/min), and *(right)* as a function of $\frac{\alpha}{\beta}$ when both forces are enabled (β = 5 μm/min, λ = 40 μm, L_{max} = 200 μm). The simulated area is $2R$ = 8 mm. ρ = 600 cells/mm^2. The structure of cells at 24 h is examined. Plots represent simulation results, and dotted curves are obtained by fitting the simulation results to the Hill function in the form of $\frac{V^q}{V^q+K^q}$ (left, center) or $\frac{K^q}{V^q+K^q}$ (right), where V is a model parameter (λ, L_{max} or α/β), q is a coefficient and and K is the critical parameter value. The values of K are *(left)* 170 μm for λ, *(center)* 90 μm for L_{max}, and *(right)* 0.008 for α/β, respectively.

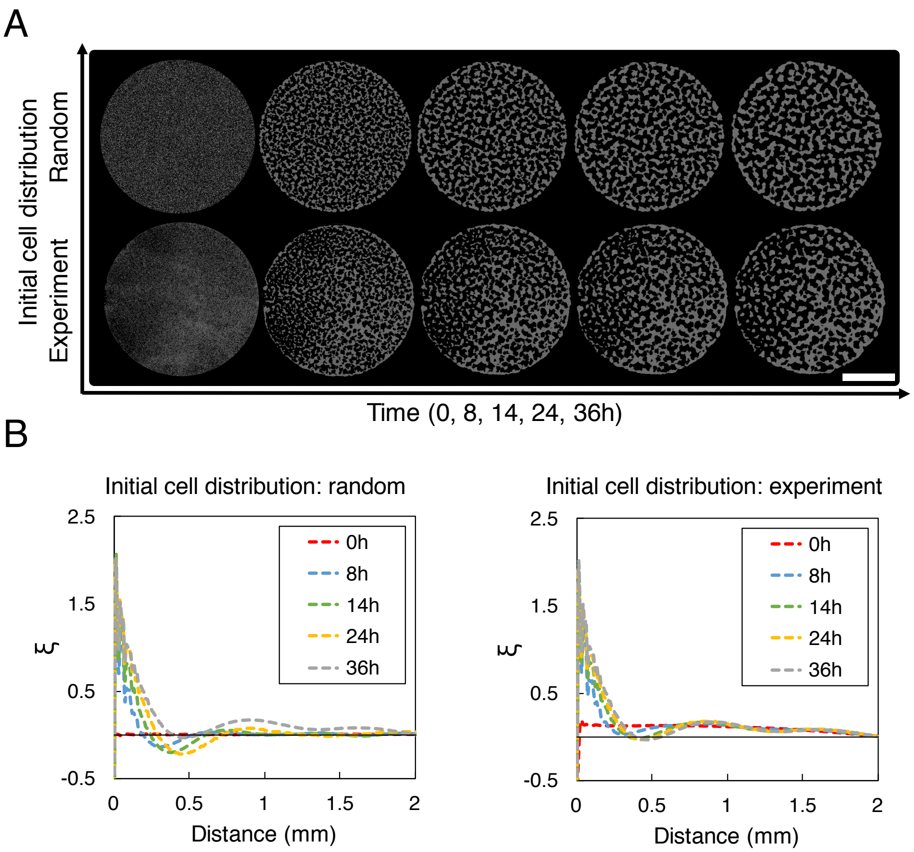

Figure S4. The structures of cells in simulations. *(A)* The structures that cells formed in simulations at 0, 8, 14, 24, and 36 h. $\alpha = 0.25$ μm/min, $\lambda = 80$ μm, $\beta = 5$ μm/min, and $L_{\max} = 200$ μm. The initial cell distribution is obtained from a Poisson point process (random) or the experiment (Fig. 4A, network-like). The cell density is approximately $\rho = 600$ cells/mm^2. Simulated area of $2R = 14$ mm in diameter is shown. Scale bar, 5 mm. *(B)* Two-point correlation function ξ of cell positions at different time instance for each of the two cases in *(A)*.

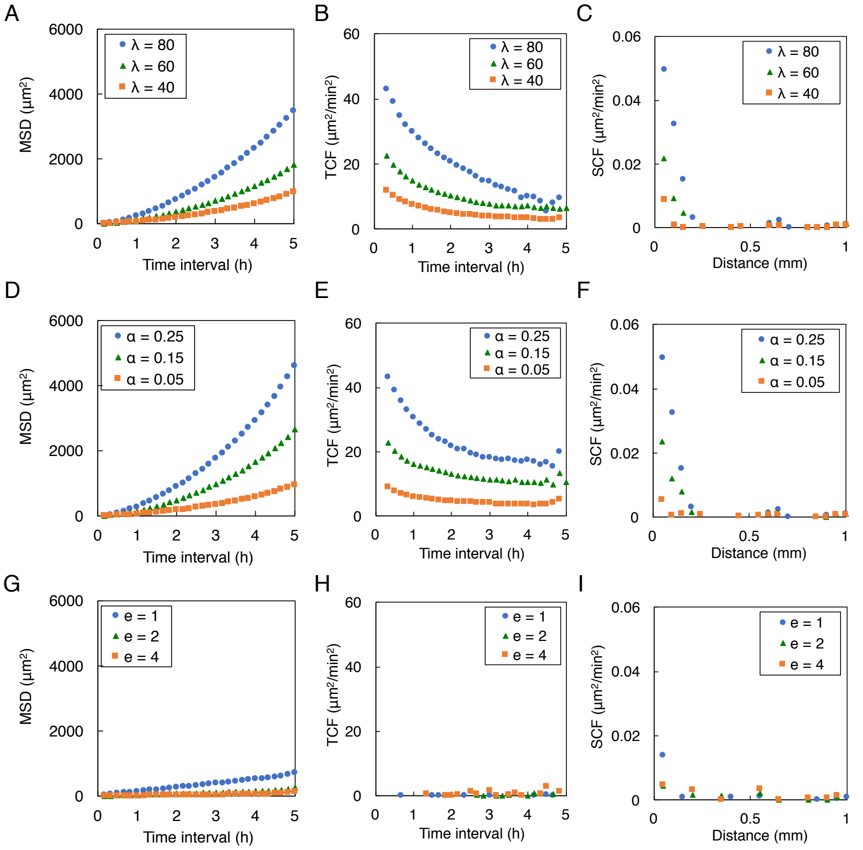

Figure S5. Simulation results. (A, D, G) Mean square displacement (MSD), (B, E, H) temporal correlation function (TCF) of cell velocities, and (C, F, I) spatial correlation function (SCF) of cell velocities. The simulated area is $2R = 4$ mm. The following parameter values were used. (A – C) $\alpha = 0.25$ μm/min, $\lambda = 40, 60, 80$ μm, $\beta = 5$ μm/min, and $L_{\max} = 200$ μm. (D – F) $\alpha = 0.05, 0.15, 0.25$ μm/min, $\lambda = 40$ μm, $\beta = 5$ μm/min, and $L_{\max} = 200$ μm. (G – I) $\alpha = \beta = 0$ μm/min, and $e = 1, 2, 4$. Default values were used for other parameters. The simulations ran over a time period of 5 h. In computing MSD, TCF and SCF, cells within $0.9 \times 2R$ in diameter from the center of the simulation area were used to reduce the effect of boundaries. See text below for details.

Experimental results (Fig. 1 C – E, gel (thick) and gel (thin)) showed that the mean square displacement (MSD), temporal correlation function (TCF) of cell velocities and spatial correlation function (SCF) of cell velocities significantly decreased when the Matrigel thickness reduced from thick (0.65 mm) to thin (0.13 mm). As we discussed in Discussion, we hypothesize that HeLa cells interacted with Matrigel, generated a remote force that propagated over the Matrigel, and coordinated their movements with other cells at distance. We also hypothesize that the Matrigel thickness determined the strength of the remote force and the distance over which the remote force travels over the Matrigel; when the Matrigel was thinner, the remote force was weaker and traveled over a shorter distance. To examine our hypotheses, we decreased the strength (α) and the characteristic length (λ) of the remote force in simulations and examined the impact of these two parameters on MSD, TCF and SCF. Simulation results (Fig. S5 A – F) show that MSD, TCF and SCF decrease as α and λ decrease (namely, the Matrigel thickness decreases), reproducing the behaviors of the decreased MSD, TCF and SCF observed in experiments when the Matrigel thickness decreased (Fig. 1 C – E, gel (thick) and gel (thin)).

Experimental results (Fig. 1 C – E, glass) also showed that, when cells were on a glass surface, MSD increased slowly, while TCF and SCF remained low over time and distance, respectively. As reported previously (28), the Langevin equation describes cell motility on the glass surface. By ignoring the inertial effects in the Langevin equation, and assuming that the remote and contact forces are negligible and, thus, the random force $\mathbf{F}^i_{\mathrm{rnd}}$ being the sole force that acts on cells, we replace our model (5) with the following model: $d\mathbf{x}_i/dt = \mathbf{F}^i_{\mathrm{rnd}}$, where $\mathbf{F}^i_{\mathrm{rnd}}$ follows a Gaussian distribution with zero mean and the variance of e^2 for each dimension. Note that the volume exclusion effect remained effective in simulations. Simulation results (Fig. S5 G – I) with the values of e varied ($e = 1, 2, 4$) show MSD, TCF and SCF exhibiting the behaviors similar to those observed in experiments (Fig. 1 C – E, glass), respectively.

Movies

Movie S1 5-h time-lapse movie when cells were plated on a glass surface (Fig. 1A and B, glass)

Movie S2 5-h time-lapse movie when cells were plated on a thin Matrigel layer [Fig. 1A and B, gel (thin)]

Movie S3 5-h time-lapse movie when cells were plated on a thick Matrigel layer [Fig. 1A and B, gel (thick)]

Movie S4 15-h time-lapse movie when cells were plated on a thick Matrigel layer (Fig. 2A and B)

Movie S5 5-h time-lapse movie showing cells forming cellular bridges (Fig. 3A)

Movie S6 Simulation of the cellular bridge formation observed in experiments (Fig. 7A)

Movie S7 Simulation of large-scale multicellualr structure formation (Fig. 8A, islands)

Movie S8 Simulation of large-scale multicellualr structure formation (Fig. 8A, network-like)

Movie S9 Simulation of large-scale multicellualr structure formation (Fig. 8A, continent)

Numerical Methods

Discrete-time and agent-based simulations were performed in this work. Time t is discretized with the time step length of Δt, and each simulation step advances time by Δt. Each cell $i \in \mathcal{N}$ is modeled as a discrete entity or an agent.

Simulations consider a set $\mathcal{N}$ of cells in a two-dimensional and circular area of the diameter of $2R$. At time t, cell i maintains its position $\mathbf{x}_i(t)$ and list $\mathcal{N}_i(t)$ containing cells that are in physical contact with cell i. Cell i also maintains its Verlet Neighbor List (VNL) $\mathcal{V}_i(t)$ containing cells that are within the distance L_{vnl} from cell i, excluding cell i itself. VNL is used to facilitate the computation of the remote force between cells. A simulation runs in the following manner:

1. Initialization Initial positions of cells, $\mathbf{x}_i(0)$ for all $i \in \mathcal{N}$, are set following a Poisson point process or based on the cell positions observed in experiments. For each cell $i \in \mathcal{N}$, its initial physical contact list $\mathcal{N}_i(0)$ and initial VNL $\mathcal{V}_i(0)$ are then set such that

- $\mathcal{N}_i(0)$ contains all cells within the contact initiation distance L_{ini} from cell i, excluding cell i itself, and
- $\mathcal{V}_i(0)$ contains all cells within the distance L_{vnl} from cell i, excluding cell i itself.

2. Main loop At every simulation time step, for each cell $i \in \mathcal{N}$, its position $\mathbf{x}_i(t)$ is updated as follows. When no cells exist within distance $L_{\min}$ from $\mathbf{x}_i(t)$,

$$\mathbf{x}_i(t+\Delta t) \quad = \quad \mathbf{x}_i(t) + \left(\mathbf{F}_i^{\mathrm{rm}}(t) + \mathbf{F}_i^{\mathrm{cn}}(t)\right)\Delta t, \tag{10}$$

and otherwise,

$$\mathbf{x}_i(t+\Delta t) \quad = \quad \mathbf{x}_i(t) + \mathbf{F}_i^{\mathrm{ex}}(t)\Delta t, \tag{11}$$

where $\mathbf{F}_i^{\mathrm{rm}}(t)$ and $\mathbf{F}_i^{\mathrm{cn}}(t)$ represent the remote and contact forces exerted on cell i at time t, respectively, and $\mathbf{F}_i^{\mathrm{ex}}(t)$ represents the effect of volume exclusion that acts on cell i at time t. These three terms $\mathbf{F}_i^{\mathrm{rm}}(t)$, $\mathbf{F}_i^{\mathrm{cn}}(t)$ and $\mathbf{F}_i^{\mathrm{ex}}(t)$ are computed as follows.

$$\mathbf{F}_i^{\mathrm{rm}}(t) \quad = \quad \alpha \sum_{\substack{j \in \mathcal{V}_i(t) \\ \left|\mathbf{x}_i(t)-\mathbf{x}_j(t)\right| \le L_{\mathrm{cut}}}} \exp\left(-\frac{\left|\mathbf{x}_j(t)-\mathbf{x}_i(t)\right|}{\lambda}\right) \frac{\mathbf{x}_j(t)-\mathbf{x}_i(t)}{\left|\mathbf{x}_j(t)-\mathbf{x}_i(t)\right|}, \tag{12}$$

$$\mathbf{F}_i^{\mathrm{cn}}(t) \quad = \quad \beta \sum_{j \in \mathcal{N}_i(t)} \max\left(\frac{\left|\mathbf{x}_j(t)-\mathbf{x}_i(t)\right| - L_{\min}}{L_{\max}-L_{\min}}, 0\right) \frac{\mathbf{x}_j(t)-\mathbf{x}_i(t)}{\left|\mathbf{x}_j(t)-\mathbf{x}_i(t)\right|}, \tag{13}$$

$$\mathbf{F}_i^{\mathrm{ex}}(t) \quad = \quad -\gamma \sum_{j \in \mathcal{N}_i(t)} \max\left(\frac{L_{\min} - \left|\mathbf{x}_j(t)-\mathbf{x}_i(t)\right|}{L_{\min}}, 0\right) \frac{\mathbf{x}_j(t)-\mathbf{x}_i(t)}{\left|\mathbf{x}_j(t)-\mathbf{x}_i(t)\right|} \tag{14}$$

Note that in calculating the remote force in Eq. 12, only the cells that are within the remote force cutoff distance L_{cut} of each other are considered. This is to avoid incurring additional computation time. In our simulations, we set $L_{\mathrm{cut}} = 10\lambda$ and $L_{\mathrm{vnl}} = 12\lambda$. However, these values may need to be decreased for computational tractability, especially when the cell density is very high.

In addition, for each cell $i \in \mathcal{N}$, its contact list $\mathcal{N}_i(t)$ is updated at every simulation time step as follows.

$$\mathcal{N}_i(t+\Delta t) \quad = \quad \mathcal{N}_i(t) \cup \mathcal{E}_i^{+}(t) \setminus \mathcal{E}_i^{-}(t), \tag{15}$$

where $\mathcal{E}_i^{+}(t)$ is the set of new cells with which cell i becomes in physical contact at time t, and $\mathcal{E}_i^{-}(t)$ is the set of cells with which cell i loses physical contact at time t. They are given below:

$$\mathcal{E}_i^{+}(t) \quad = \quad \left\{ j : j \in \mathcal{V}_i(t) \setminus \mathcal{N}_i(t), \left|\mathbf{x}_i - \mathbf{x}_j\right| \le L_{\mathrm{ini}}, i \ne j \right\}, \tag{16}$$

$$\mathcal{E}_i^{-}(t) \quad = \quad \left\{ j : j \in \mathcal{N}_i(t), \left|\mathbf{x}_i - \mathbf{x}_j\right| > L_{\max}, i \ne j \right\}. \tag{17}$$

Further, for each cell $i \in \mathcal{N}$, we update at every 100 simulation time steps its $\mathcal{V}_i(t)$ such that $\mathcal{V}_i$ contains all cells within L_{vnl} from cell i, excluding cell i itself. $\mathcal{V}_i(t)$ is given as follows.

$$\mathcal{V}_i(t) \quad = \quad \left\{ j : j \in \mathcal{N}, \left|\mathbf{x}_i(t) - \mathbf{x}_j(t)\right| < L_{\mathrm{vnl}}, i \ne j \right\} \tag{18}$$

Note that $\mathcal{V}_i(t)$ is not updated at every simulation time step to avoid incurring additional computation time.

Obtaining Two Point Correlation Functions

We computed the two-point correlation function $\xi(r)$ in Eq. 4, also shown below,

$$\mathrm{d}N(r) = 2\pi r \mathrm{d}r \rho \left(1 + \xi\left(r\right)\right), \tag{19}$$

in the following manner.

The left-hand side of Eq. 19, $\mathrm{d}N(r)$, represents the number of cells located at distance r to $\mathrm{d}r$ from a randomly selected cell in the circular area of experiments or simulations. Computing $\mathrm{d}N(r)$ requires the spatial positions of all cells. To compute $\mathrm{d}N(r)$ from experimental results, we used the spatial positions of cells identified from microscopy images of cells. To compute $\mathrm{d}N(r)$ from simulations, we used the spatial positions of all cells tracked in simulations. Once $\mathrm{d}N(r)$ is computed, $\xi(r)$ can be obtained using Eq. 19.

In computing $\mathrm{d}N(r)$, and thus, $\xi(r)$, in Eq. 19, we reduced the effect of boundaries of the Matrigel area in experiments and of the simulated area as follows. We first selected a subset $\mathcal{N}'$ of cells such that each cell $i \in \mathcal{N}'$ is located within 0.4 mm from the center of the area when $R = 4$ mm or 2.8 mm from the center of the area when $R = 7$ mm. We then counted $\mathrm{d}N(r)$, varying r from 0 to 2.8 mm with an increment of $\mathrm{d}r = 0.02$ mm as follows:

$$\mathrm{d}N(r) = \frac{1}{|\mathcal{N}'|} \sum_{i \in \mathcal{N}'} \left| \left\{ j : j \in \mathcal{N}, r < d_{i,j} \leq r + \mathrm{d}r \right\} \right|, \tag{20}$$

where $d_{i,j}$ is the distance between cells i and j.
